# Atmospheric sulfate aerosol formation enhanced by interfacial anions

**DOI:** 10.1093/pnasnexus/pgaf058

**Published:** 2025-02-24

**Authors:** Gehui Wang, Si Zhang, Can Wu, Tong Zhu, Xinbei Xu, Shuangshuang Ge, Haitao Sun, Zhenrong Sun, Jiaxin Wang, Yuemeng Ji, Jian Gao, Yanqin Ren, Hong Li, Fang Zhang, Yuan Wang, John H Seinfeld

**Affiliations:** School of Geographic Sciences, Key Lab of Geographic Information Science of the Ministry of Education, East China Normal University, Shanghai 200241, China; School of Geographic Sciences, Key Lab of Geographic Information Science of the Ministry of Education, East China Normal University, Shanghai 200241, China; School of Geographic Sciences, Key Lab of Geographic Information Science of the Ministry of Education, East China Normal University, Shanghai 200241, China; School of Chemistry and Molecular Engineering, East China Normal University, Shanghai 200241, China; School of Geographic Sciences, Key Lab of Geographic Information Science of the Ministry of Education, East China Normal University, Shanghai 200241, China; School of Geographic Sciences, Key Lab of Geographic Information Science of the Ministry of Education, East China Normal University, Shanghai 200241, China; State Key Laboratory of Precision Spectroscopy, School of Physics and Electronic Sciences, East China Normal University, Shanghai 200241, China; State Key Laboratory of Precision Spectroscopy, School of Physics and Electronic Sciences, East China Normal University, Shanghai 200241, China; School of Environmental Science and Engineering, Guangdong University of Technology, Guangzhou 510006, China; School of Environmental Science and Engineering, Guangdong University of Technology, Guangzhou 510006, China; State Key Laboratory of Environmental Criteria and Risk Assessment, Chinese Research Academy of Environmental Sciences, Beijing 100012, China; State Key Laboratory of Environmental Criteria and Risk Assessment, Chinese Research Academy of Environmental Sciences, Beijing 100012, China; State Key Laboratory of Environmental Criteria and Risk Assessment, Chinese Research Academy of Environmental Sciences, Beijing 100012, China; School of Civil and Environmental Engineering, Harbin Institute of Technology (Shenzhen), Shenzhen 518055, China; Department of Earth System Science, Stanford University, Stanford, CA 94305, USA; Division of Chemistry and Chemical Engineering, California Institute of Technology, Pasadena, CA 91125, USA

**Keywords:** air pollution, China haze, sulfate aerosols, interface chemistry, aerosol kinetics

## Abstract

Heterogeneous oxidation of SO_2_ by NO_2_ on aerosols has recently been found to be one of the major formation pathways of sulfate in the polluted troposphere, but the chemical mechanisms and kinetics remain uncertain. By combining lab experiments, theoretical chemistry calculations, and field measurements, here we show that the SO_2_ oxidation by NO_2_ is critically dependent on anions at the air–aerosol aqueous interface. The reaction rate of NO_2_ with ${\mathrm{HSO}}_{3}^{-}$ (1.1 × 10^8^–1.6 × 10^9^ M^−1^ s^−1^) is more than four orders of magnitude larger than the traditionally held value for the bulk phase due to the abundant occurrence of chloride, nitrate, and carboxylic anions at the air–aqueous interface, which remarkably accelerates sulfate formation during China haze periods by enhancing the uptake of NO_2_ through interfacial electrostatic attraction. Atmospheric models not accounting for this aerosol interfacial process likely produce major misrepresentations of tropospheric sulfate aerosols under polluted conditions.

**Significance Statement**

A high level of sulfate still frequently occurs in haze periods in China, but the formation mechanism remains unclear. From atmospheric measurement in Beijing, laboratory experiments, and quantum chemical simulation, we show that the heterogeneous oxidation of SO_2_ by NO_2_ in aerosol aqueous phase is the predominant pathway of sulfate formation during Chinese haze periods, which is significantly enhanced by anions at the air–aqueous interface through an electrostatic attraction with NO_2_. Our work reveals that air–aqueous interfacial chemistry is a key role in secondary aerosol formation in real atmosphere and should be accounted for models.

## Introduction

Sulfate is one of the major components of atmospheric aerosols and profoundly affects climate, human health, and the environment (1–3). Tropospheric sulfate is largely formed from heterogeneous reactions of SO_2_ with oxidants such as O_3_, H_2_O_2_, and O_2_ catalyzed by transition metal ions (TMIs, mainly Fe^3+^ and Mn^2+^) in aerosols, fogs, and cloud droplets (2, 4–7). However, the rapid formation of sulfate in China haze periods has often been underestimated by numerical models, suggesting some unknown sulfate formation mechanisms (8, 9). Recently, a few studies have proposed that heterogeneous oxidation of SO_2_ by NO_2_ with neutralization by NH_3_ under humid conditions is a major formation pathway of sulfate during winter haze periods in China (1, 7, 10–16), but these studies are controversial regarding the role of NO_2_ in the SO_2_ oxidation process. Some researchers have proposed that the oxidation of SO_2_ by NO_2_ in the aerosol aqueous phase produces sulfate and HONO, and HONO subsequently evaporates into the gas phase (Eqs. 1 and 2) (1, 7, 17). In contrast, others presume that the dissolved SO_2_ is oxidized into ${\mathrm{SO}}_{4}^{2-}$ not only by NO_2_ (Eq. 1) but also by ${\mathrm{NO}}_{2}^{-}$, and the latter is reduced into N_2_O and subsequently evaporates into the gas phase (Eq. 3) (10, 13, 18). Such controversial results suggest that some fundamental processes governing the reaction of SO_2_ with NO_2_ in the troposphere are still not understood. Therefore, revealing the mechanisms and kinetics is necessary because SO_2_, NO*_x_*, and their secondary products, e.g. sulfate, nitrate, and ozone, are the major pollutants in the troposphere.


(1) $$\begin{array}{rl} 2NO_{2}(\mathrm{aq}) & +S(\mathrm{IV})(\mathrm{aq})+H_{2}O\to 2H^{+\;}(\mathrm{aq})\\ & +{{2\mathrm{NO}}_{2}}^{-}(\mathrm{aq})+S(\mathrm{VI})(\mathrm{aq}) \end{array}$$

(2) $$H^{+\;}(\mathrm{aq})+{{\mathrm{NO}}_{2}}^{-}(\mathrm{aq})\to \mathrm{HONO}(g)$$

(3) $${{2\mathrm{NO}}_{2}}^{-}(\mathrm{aq})+2S(\mathrm{IV})(\mathrm{aq})\to N_{2}O(g)+2S(\mathrm{VI})$$

(4) $$2NO_{2}+H_{2}O(\mathrm{aq})\to \mathrm{HONO}+\mathrm{HN}O_{3}$$

To date, studies on the heterogeneous formation of sulfate through SO_2_ oxidation in aerosols, fogs, and clouds have only focused on the reaction processes in the bulk phase and deemed that the bulk-phase acidity (pH) is a crucial factor controlling the SO_2_ oxidation pathways and sulfate production in the global atmosphere (10, 14, 19–22). In the past decade, a number of studies have found that ion distributions at the air–water interface of an electrolyte solution are different from those in the bulk phase. Some polarizable anions, such as Cl^−^, Br^−^, I^−^, ${\mathrm{NO}}_{3}^{-}$, and ${\mathrm{HCO}}_{3}^{-}$, preferably stay near the most top surface, while some cations, such as Na^+^ and Ca^2+^, and divalent anions, such as ${\mathrm{SO}}_{4}^{2-}$ and ${\mathrm{CO}}_{3}^{2-}$, stay near the bulk phase (23–26). Such different distributions can result in anions being enriched in the air–aqueous interface (27, 28), and thus may significantly affect the reactions of gaseous pollutants with atmospheric aerosols, fogs, and cloud droplets. However, direct field evidence on such an interfacial effect is lacking, and thus, the involved atmospheric reactions are unknown because compositions of the air–aqueous interface in the atmosphere cannot be measured directly.

Here, we used a laboratory smog chamber (Fig. S1) to mimic the impact of the air–aqueous interfacial ions on the heterogeneous oxidation of SO_2_ by NO_2_ in preexisting aerosols during haze periods in China by exposing a series of inorganic and organic seeds to SO_2_, NO_2_, and NH_3_ under humid conditions. Both gas- and aerosol-phase species were comprehensively monitored online by using a series of sophisticated instruments and simulated by using state-of-the-art molecular dynamics (MD) models and quantum chemical calculations to explore the reaction process of SO_2_ with NO_2_. Based on the field observations, laboratory chamber experiments, and MD simulations, we found a key role of air–aqueous interfacial anions in sulfate formation process in Beijing by investigating the winter haze chemistry.

## Results

### Distinct behavior of SO_2_ oxidation at the air–aqueous interface

**Fig. 1.**
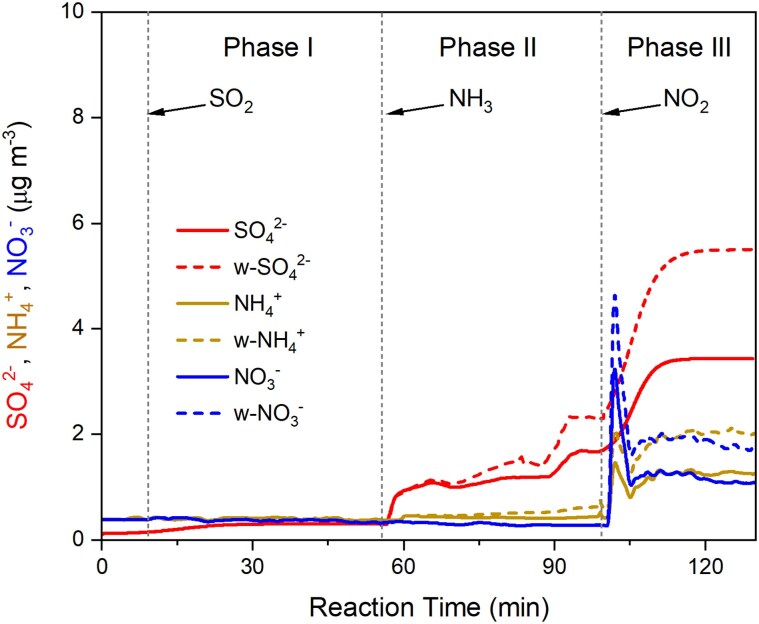
Changes in compositions of particles in the chamber during the exposure of NaCl seeds to SO_2_, NH_3_, and NO_2_ under 90% RH conditions. The figure shows the time evolution of aerosol-phase (${\mathrm{SO}}_{4}^{2-}$, ${\mathrm{NH}}_{4}^{+}$, ${\mathrm{NO}}_{3}^{-}$, and wall-loss-corrected ${\mathrm{SO}}_{4}^{2-}$ ($w\text{-}{\mathrm{SO}}_{4}^{2-}$), ${\mathrm{NH}}_{4}^{+}$ ($w\text{-}{\mathrm{NH}}_{4}^{+}$), and ${\mathrm{NO}}_{3}^{-}$ ($w\text{-}{\mathrm{NO}}_{3}^{-}$) species for a typical NaCl aerosol seed experiment under 80 ppb NH_3_ (g) conditions. $w\text{-}{\mathrm{SO}}_{4}^{2-}$, $w\text{-}{\mathrm{NH}}_{4}^{+}$, and $w\text{-}{\mathrm{NO}}_{3}^{-}$ concentrations were derived by correcting ${\mathrm{SO}}_{4}^{2-}$, ${\mathrm{NH}}_{4}^{+}$, and ${\mathrm{NO}}_{3}^{-}$ concentrations using a wall-loss rate before adding NH_3_ into the chamber (see Materials and methods for more details).

To investigate the heterogeneous oxidation of SO_2_ by NO_2_ on preexisting aerosols in urban atmosphere, we exposed different types of seeds on a polydisperse mode including NaCl, NH_4_NO_3_, (NH_4_)_2_SO_4_, a mixture of (NH_4_)_2_SO_4_ and NaCl, oxalic acid, and sucrose, which are typical components of tropospheric aerosols (29, 30), to the same levels of SO_2_ (600 ppb), NO_2_ (600 ppb), and NH_3_ (80 and 190 ppb) under 90% relative humidity (RH) conditions, respectively. All the seed solution was added with ethylenediaminetetraacetic acid (EDTA) before being nebulized into the chamber to remove a possible impact of TMIs. Figure 1 shows the variations in concentrations of sulfate, nitrate, and ammonium in the chamber during the exposure of NaCl seeds. When SO_2_ was introduced, a very small amount of sulfite was formed on the NaCl seeds (phase I in Fig. 1). The sulfate increased to about 1.5 μg m^−3^ after NH_3_ was introduced (phase II in Fig. 1). Such a small amount of sulfate formed in the absence of NO_2_ is most likely caused by a reaction between SO_2_ and impurities of peroxides in the solution and the increased dissolved S(IV) (15). After NO_2_ was further introduced, S(IV) was oxidized into S(VI) and a sharp increase of 4.0 μg m^−3^ sulfate was detected (phase III in Fig. 1), suggesting a rapid formation of sulfate on the NaCl seeds via heterogeneous oxidation of SO_2_ by NO_2._ The reactive uptake coefficients of SO_2_ in the three stages were 2.5 × 10^−7^ (unary uptake, SO_2_ only, phase I in Fig. 1), 1.5 × 10^−6^ (co-uptake, SO_2_ and NH_3_, phase II in Fig. 1), and 2.6 × 10^−5^ (trinary uptake, SO_2_, NH_3_, and NO_2_, phase III in Fig. 1), respectively, indicating an enhanced interaction of SO_2_ with NO_2_ in the presence of NH_3_. Such a SO_2_ uptake was greater under 190 ppb NH_3_ conditions (Tables S1 and S2), because NH_3_ reduces the aerosol acidity and makes more S(IV) available at the interface. Similar results were also observed for oxalic acid, NH_4_NO_3_, and (NH_4_)_2_SO_4_/NaCl mixture seeds, respectively (Fig. S2A–C, and Tables S1 and S2). However, no additional sulfate was formed on either (NH_4_)_2_SO_4_ or sucrose seeds during the exposure (Fig. S2D and E). Such distinct differences in sulfate formation should be ascribed to the properties of seeded particles rather than the addition of EDTA into the seeds, because all the seeds contained the same amount (2 mM) of EDTA. Figure S3 compares the sulfate production rate on NaCl seeds with a different size. It can be seen that both *γ*, which is the uptake coefficient of SO_2_, and $R_{{\mathrm{SO}}_{4}^{2-}}$/SA, which is the sulfate formation rate ($R_{{\mathrm{SO}}_{4}^{2-}}$) normalized by the surface area (SA) of seeds, are ∼50% higher on NaCl seeds with a 60-nm diameter than on NaCl seeds with a 100-nm diameter. Such a dependence of sulfate production rate on seed particle size is in agreement with the experimental results reported by Liu and Abbatt (15) and suggests that the heterogeneous oxidation of SO_2_ by NO_2_ proceeds at the aerosol surface.

**Fig. 2.**
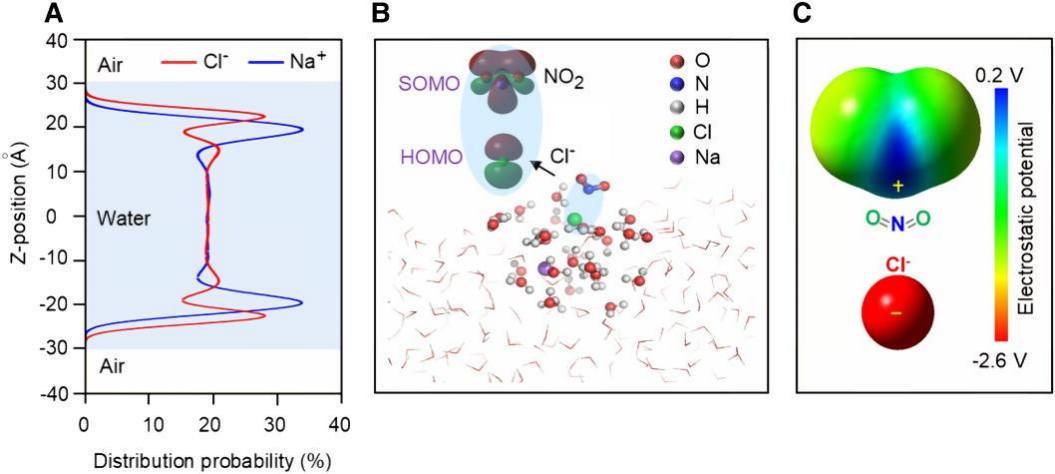
Multiscale simulations of the NO_2_-Cl^−^ interaction at the air–aqueous interface. A) MD simulation of the distribution of ions at the air–water interface of a 2-M NaCl solution (the simulation was run in the NVT (NVT is the canonical ensemble, which is a statistical mechanism state distribution where the number of particles (N), volume (V) and temperature (T) are constant) ensemble for 200 ns, and the system temperature was kept at 298 K). B) A typical snapshot of the absorption of NO_2_ on a wetted NaCl aerosol. The frontier orbitals of Cl^−^ HOMO and NO_2_ SUMO were also calculated. C) Electrostatic potential distributions of NO_2_ and Cl^−^.

On the contrary to the traditional Debye–Hückel theory of bulk aqueous electrolytes, a number of studies found that in the air–water interface of an electrolyte solution, some polarizable ions, such as halogen ions (Cl^−^, Br^−^, and I^−^), ${\mathrm{NO}}_{3}^{-}$, ${\mathrm{HCO}}_{3}^{-}$, carboxylate, and ${\mathrm{NH}}_{4}^{+}$, stay closer to the surface, while other ions, such as Na^+^, K^+^, ${\mathrm{SO}}_{4}^{2-}$, and ${\mathrm{CO}}_{3}^{2-}$, stay deeper in the bulk phase (31, 32). Thus, the air–aqueous interfaces of wetted NaCl and oxalate seeds are enriched with anions, while that of (NH_4_)_2_SO_4_ seeds are enriched with cations. We assume that the anions at the air–aqueous interface of an aerosol may greatly promote the transport of NO_2_ from the gas phase into the aqueous phase and thus enhance SO_2_ oxidation by NO_2_. To elucidate this effect, we used an MD model to simulate the distribution of ions in a NaCl solution droplet with a concentration (2 M NaCl) similar to that in our chamber experiments (33). As shown in Fig. 2A, Na^+^ has a high distribution at a distance of ∼11 Å from the water surface, while Cl^−^ has the largest distribution at a distance of ∼8 Å from the water surface, which means that Cl^−^ ions are more accessible to the air–aqueous surface (34). At deeper positions, the concentrations of both ions are nearly the same (Fig. 2A). The layered Na^+^ and Cl^−^ can form an electric field directed toward the water surface, which is the same as the direction of the dipole moment of NO_2_ and thus efficiently promotes the adsorption of NO_2_. When NO_2_ moves from the air to the surface of the water slab, it may even directly collide with Cl^−^ ions at the surface (Fig. 2B). Figure 2C shows the electrostatic potential of the NO_2_–Cl^−^ interaction calculated by the quantum chemical method. It can be seen that the negatively charged Cl^−^ ion has a strong attraction to the partially positively charged N atom. In fact, a *σ* bond could be formed between the single unoccupied molecular orbital (SUMO) of NO_2_ and the highest occupied molecular orbital (HOMO) of Cl^−^ (Fig. 2B), which therefore significantly enhances NO_2_ uptake by the wetted NaCl aerosols.

Because at the air–water interface of a solution ${\mathrm{NH}}_{4}^{+}$ ions preferably stay near the surface with the counterpart ${\mathrm{SO}}_{4}^{2-}$ ions locating deeper in the bulk phase (35, 36), and sucrose is a nonionizable organic compound, the air–water interfaces of (NH_4_)_2_SO_4_ and sucrose seeds in the chamber lack anions. Therefore, NO_2_ molecules cannot effectively dissolve into the aerosol aqueous phase due to their poor solubility (2). As a result, SO_2_ cannot be efficiently oxidized by NO_2_, and thus, no sulfate was formed on (NH_4_)_2_SO_4_ and sucrose seeds unless NaCl was added to the (NH_4_)_2_SO_4_ solution (Fig. S2C–E). Hua et al. (37) found that ${\mathrm{NO}}_{3}^{-}$ is more accessible than ${\mathrm{NH}}_{4}^{+}$ to the air–water surface. Mahiuddin et al. (38) investigated the surface behavior of aqueous solutions of diacids, including oxalic acid using MD model, and found all these acids exhibit a significant propensity for the air–water interface. Enami et al. (39) later reported that dicarboxylic acids at the surface largely exist as monoanion and undissociated forms. Thus, like NaCl seeds, the air–aqueous interface of NH_4_NO_3_ and oxalic acid seeds is also enriched with anions, which effectively trapped NO_2_ and resulted in a rapid formation of sulfate after NO_2_ was introduced (Fig. S2B, and Tables S1 and S2).

### Oxidation pathway of SO_2_ by NO_2_

To clarify the oxidation pathway of SO_2_ by NO_2_, we exposed NaCl seeds to NO_2_, SO_2_, and NH_3_ in an order being different from that in Fig. 1. As seen in Fig. S4, at phase I, NO_2_ molecules disproportionate on the wetted seeds (Eq. 4) (40, 41), thus, ∼8.0 μg m^−3^ of ${\mathrm{NO}}_{3}^{-}$ in the chamber was detected. After SO_2_ was added into the chamber, the heterogeneous oxidation of SO_2_ with NO_2_ proceeded at the air–aqueous interface of the NaCl seeds. At this moment, only a small amount of sulfate was detected (Fig. S4, phase II), because the particles became very acidic due to the formation of sulfuric acid, which prevented further dissolution of SO_2_ and thus limited the availability of S(IV). After NH_3_ was introduced into the chamber, both ${\mathrm{SO}}_{4}^{2+}$ and ${\mathrm{NH}}_{4}^{+}$ significantly increased due to the neutralization of NH_3_ (Fig. S4, phase III), but ${\mathrm{NO}}_{3}^{-}$ concentration was almost constant as that in phase I, suggesting that NH_3_ may accelerate NO_2_ hydrolysis rate (42) but does not change the reaction equilibrium. During the whole process, no N_2_O or NO was produced. Moreover, after SO_2_ and NH_3_ were injected into the chamber, the increase in HONO concentration was stoichiometrically equal to the decreased NO_2_ concentration in the absence and presence of NaCl seeds (Fig. S5). These results clearly show that Eqs. 1 and 2, rather than Eq. 3, are the reaction pathways of SO_2_ oxidation by NO_2_. The ISORROPIA-II thermodynamic model calculation results show that the pH values of NaCl particles in the chamber after the reaction were ∼5.0 (Tables S1 and S2). Our previous work revealed that under such moderate acidic conditions, the gas-phase HONO(g) concentration in the chamber is 2–3 orders of magnitude higher than that in the aerosol liquid phase due to the high volatility of HONO and the high specific SA of aerosols (17). Therefore, in the chamber, the HONO formed in the aerosol aqueous phase quickly evaporated into the gas phase, resulting in very minor amounts of HONO and ${\mathrm{NO}}_{2}^{-}$ remaining in the aqueous phase (17). Consequently, oxidation of S(IV) by dissolved HONO (aq) or ${\mathrm{NO}}_{2}^{-}$ was negligible, and no N_2_O(g) in the chamber was detected (Fig. S4).

**Fig. 3.**
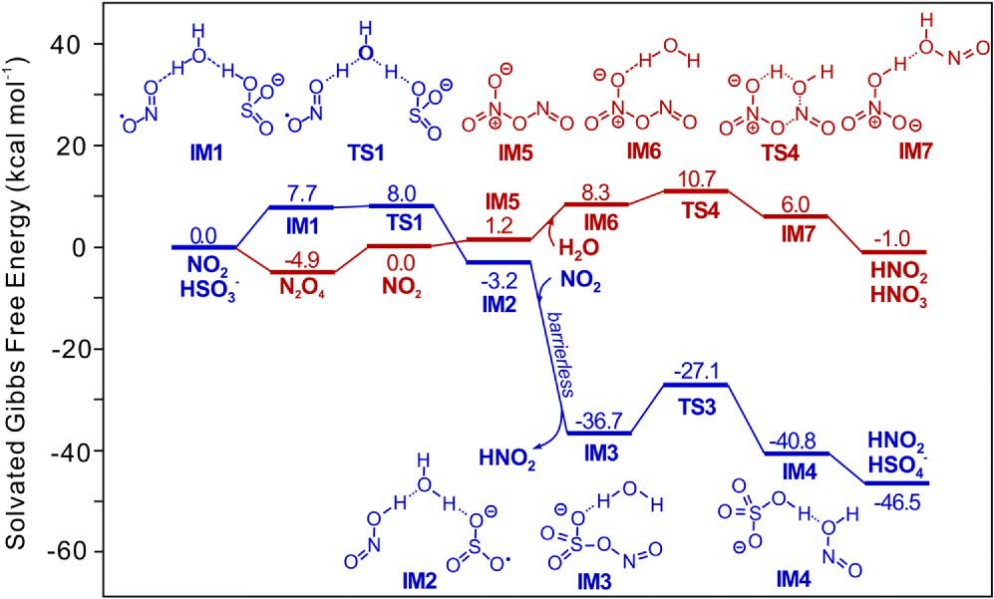
Reaction pathways of NO_2_ with ${\mathrm{HSO}}_{3}^{-}$ and NO_2_ disproportionation. All energies labeled are the Gibbs free energy calculated at the PBE0-D3/def2-TZVPD level with SMD solvation in water.

To further clarify the reaction pathway of SO_2_ with NO_2_ in the aerosol aqueous phase, we carried out a computational study to investigate the reactions of SO_2_ and NO_2_ in water by using density functional theory (43–45) and compare the NO_2_ disproportionation pathway with the redox between NO_2_ and SO_2_ (${\mathrm{HSO}}_{3}^{-}$). As shown in Fig. 3, the pathway of disproportionation starts from a NO_2_ dimer, in which the optimal pathway has a six-membered ring transition state TS4 as the rate-determining state. Relative to a NO_2_ dimer, TS4 requires a 10.7-kcal mol^−1^ activation free energy. Alternatively, at higher concentration, the disproportionation pathway can start from the most stable N_2_O_4_, which has a lower Gibbs free energy of −4.9 kcal mol^−1^, making the pathway behave as a first order reaction with an activation Gibbs free energy of 15.6 kcal mol^−1^. For the pathway of NO_2_ reduction by ${\mathrm{HSO}}_{3}^{-}$, one NO_2_ molecule and one ${\mathrm{HSO}}_{3}^{-}$ ion can be connected via hydrogen bonding with solvent water, forming intermediate IM1. An asynchronous concerted dual hydrogen atom transfer process can smoothly connect IM1 to IM2 via TS1. Relative to the substrate, TS1 has a Gibbs free energy of 8.0 kcal mol^−1^, which is 2.7 kcal mol^−1^ lower than that of NO_2_ disproportionation, approximately corresponding to a 100-fold difference in conversion rate of NO_2_. Through TS1, one molecule HONO and a ${\mathrm{SO}}_{3}^{-}$ radical anion were generated, which are still connected through a water molecule. After IM2 formation, another molecule of NO_2_ radical can readily react with ${\mathrm{SO}}_{3}^{-}$ radical through radical–radical coupling. This process is barrierless and highly spontaneous, giving the S–O-bonded intermediate IM3. IM3 can then react with water through a six-membered ring transition state TS3 to give IM4, which subsequently dissociate into ${\mathrm{HSO}}_{4}^{-}$ and another molecule of HONO.

### Kinetics of SO_2_ oxidation by NO_2_

In the chamber, pH values of the aqueous phase of seed particles are 3.5–5.0 (Tables S1 and S2), which are similar to those observed in Beijing winter haze periods (Table S3). Under such moderate acidic conditions, NO_2_ reacts mostly with ${\mathrm{HSO}}_{3}^{-}$ rather than ${\mathrm{SO}}_{3}^{2-}$ (2, 46). Thus, the formation rate of sulfate can be expressed as follows (see the details in SI Appendix S1):


(5) $$\frac{{d[\mathrm{SO}}_{4}^{2-}]}{dt}=\left(\frac{k_{{\mathrm{NO}}_{2}^{+}{\mathrm{HSO}}_{3}^{-}}}{\left[H^{+\;}\right]}\right)k_{a1}H_{SO_{2}}P_{SO_{2}}H_{NO_{2}}P_{NO_{2}}$$

where d[${\mathrm{SO}}_{4}^{2-}$] is the molar concentration of sulfate during the reaction time of d*t*. $k_{{\mathrm{NO}}_{2}^{+}{\mathrm{HSO}}_{3}^{-}}$ is the reaction rate constant of Eq. 1. [H^+^] is the molar concentration of hydrogen ions in the aqueous phase of seed particles, which was calculated by using the ISORROPIA-II model. $k_{a1}$ is the dissociation constant of ${\mathrm{HSO}}_{3}^{-}$. $H_{SO_{2}}$ and $H_{NO_{2}}$ are the Henry's law constants of SO_2_ and NO_2_. $P_{SO_{2}}$ and $P_{NO_{2}}$ are the averaged partial pressures of SO_2_ and NO_2_ in the chamber during the reaction time of d*t* (2, 15).


(6) $$k_{\exp}=\frac{({d[\mathrm{SO}}_{4}^{2-}])/dt}{k_{a1}H_{SO_{2}}P_{SO_{2}}H_{NO_{2}}P_{NO_{2}}}=\frac{k_{{\mathrm{NO}}_{2}^{+}{\mathrm{HSO}}_{3}^{-}}}{\left[H^{+\;}\right]}$$

(7) $$k_{{\mathrm{NO}}_{2}^{+}{\mathrm{HSO}}_{3}^{-}}=k_{\exp}\;[H^{+\;}]$$

**Fig. 4.**
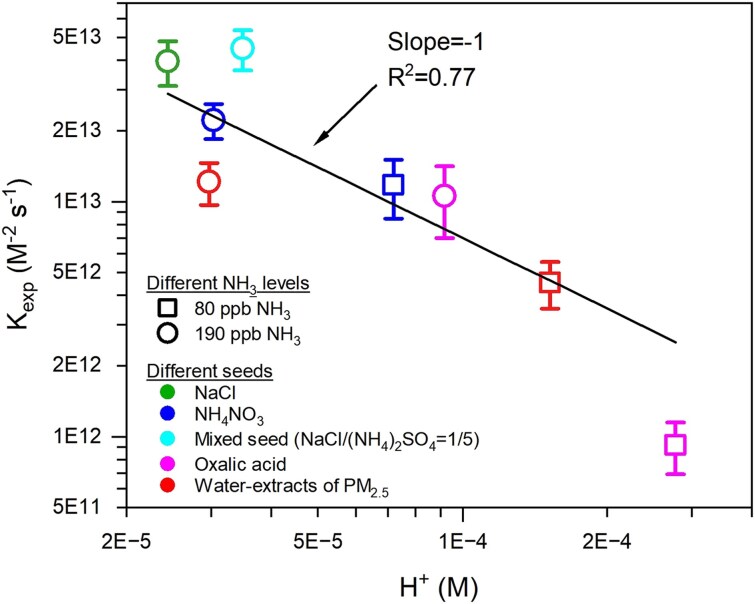
$K_{\exp}$ as a function of hydrogen ion activity.

Based on the Eq. 5, the total reaction rate $k_{\exp}$ of SO_2_ with NO_2_ in the chamber, i.e. Eq. 6, can be further expressed as the Eq. 7. By plotting a log–log relationship of $k_{\exp}$ with [H^+^], we found that $k_{\exp}$ robustly linearly correlated with hydrogen ion activity during the reaction of SO_2_ with NO_2_ in the chamber with a slope of −1 (*R*^2^ = 0.77, Fig. 4), again demonstrating that the reaction of NO_2_ with ${\mathrm{HSO}}_{3}^{-}$ rather than with ${\mathrm{SO}}_{3}^{2-}$ is the dominant formation pathway of sulfate in the chamber. We further calculated the effective rate constant $k_{{\mathrm{NO}}_{2}^{+}{\mathrm{HSO}}_{3}^{-}}$ of oxidation of SO_2_ by NO_2_ in the chamber by using the Eq. 7 (2, 15). As seen in Tables S1 and S2, $k_{{\mathrm{NO}}_{2}^{+}{\mathrm{HSO}}_{3}^{-}}$ values ranged from 1.1 × 10^8^ to 1.6 × 10^9^ M^−1^ s^−1^ under the two levels of NH_3_ conditions, which are over 4 orders of magnitude higher than those (1.5 × 10^4^–1.4 × 10^5^ M^−1^ s^−1^) obtained for bulk solutions (46, 47), further revealing that SO_2_ oxidation by NO_2_ is enhanced by anions at the air–aqueous interface.

## Discussion

**Fig. 5.**
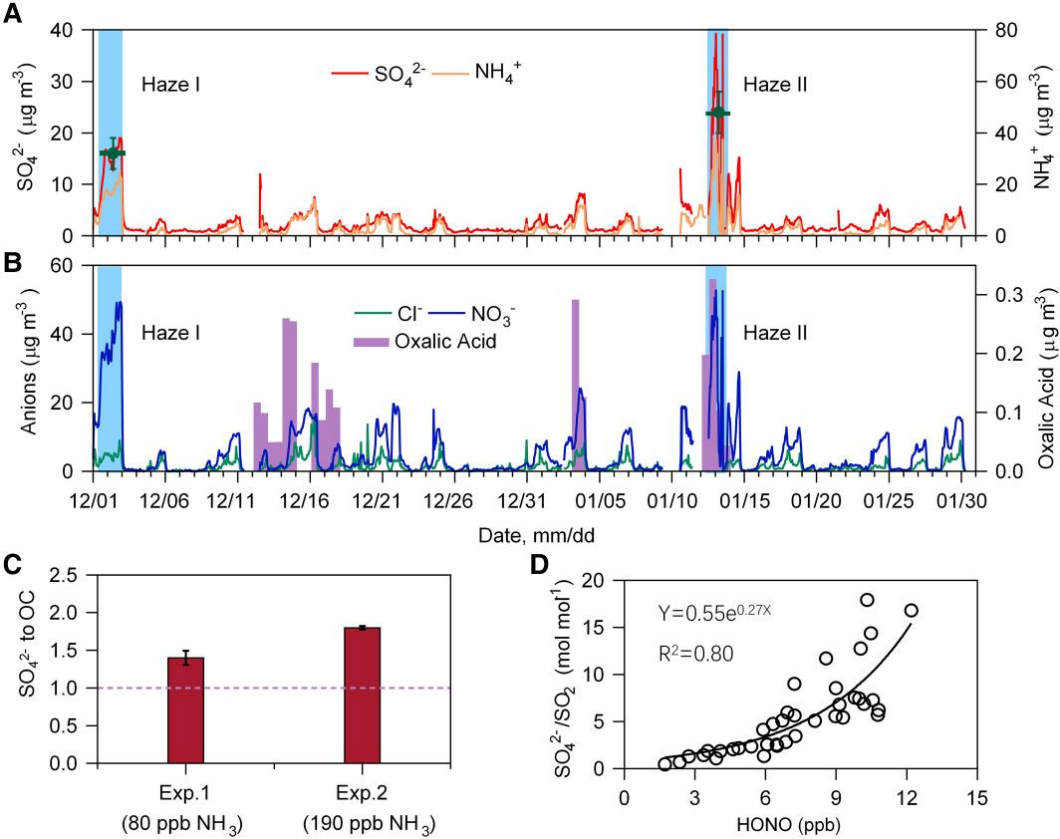
Sulfate formation in China haze episodes. A and B) Temporal variations in the concentrations of ${\mathrm{NH}}_{4}^{+}$, ${\mathrm{SO}}_{4}^{2-}$, ${\mathrm{NO}}_{3}^{-}$, Cl^−^, and oxalic acid in PM_2.5_ in Beijing during the 2018 winter campaign. Blue shadows indicate a haze event with a daily PM_2.5_ larger than 200 μg m^−3^, and the short lines in (A) are the average concentrations of ${\mathrm{SO}}_{4}^{2-}$ in PM_2.5_ with the SD in the two haze periods. C) Concentration ratio of ${\mathrm{SO}}_{4}^{2-}$ to OC in the chamber after exposing the water extracts of PM_2.5_ collected in Beijing during the 2018 Haze II event to SO_2_ (600 ppb), NO_2_ (600 ppb), and NH_3_ (80 and 190 ppb) under 90% RH conditions for 2.5 h. D) Molar ratio of ${\mathrm{SO}}_{4}^{2-}$ to SO_2_ as a function of HONO(g) concentration in Beijing during nighttime (16:00–8:00) in the Haze I and Haze II episodes.

To recognize whether such an interfacial effect is important for sulfate formation in the real troposphere, we investigated the aerosol chemistry in Beijing, China, during winter 2018. As shown in Fig. S6, the daily concentration of PM_2.5_ in Beijing during the campaign frequently exceeded 75 μg m^−3^, the second grade National Air Quality Standard, among which two heavy haze events occurred in the city with a daily PM_2.5_ level >200 μg m^−3^. During the two haze events, sulfate sharply increased to 20 and 39 μg m^−3^ (Fig. 5A, Table S3), with an uptake coefficient ($\gamma_{SO_{2}}$) of SO_2_ of 7.0 ± 1.2 × 10^−5^ in Haze I and 9.5 ± 0.2 × 10^−5^ in Haze II (Table S4), indicating an efficient heterogeneous oxidation of SO_2_. The sum of the concentrations of ${\mathrm{NO}}_{3}^{-}$ and Cl^−^ accounted for 20 and 17% of PM_2.5_ in the two haze periods. In addition, over 0.3 μg m^−3^ oxalic acid in PM_2.5_ was detected in Beijing during the Haze II event (Fig. 5B). We assumed that these abundant chloride, nitrate, and carboxylic anions accumulated in the air–aqueous interface of aerosols in the haze development process and resulted in the rapid formation of sulfate by enhancing the uptake of NO_2_.

To corroborate the above assumption, we mimicked sulfate formation in Beijing during haze episodes by exposing particles that had been atomized from water extracts of PM_2.5_ samples collected during the Haze II period to SO_2_, NO_2_, and NH_3_ under 90% RH conditions. As shown in Fig. 5C, a significant increase in the mass ratio of sulfate to OC was observed after exposure, with values of 1.4 ± 0.1 and 1.8 ± 0.02 under 80 and 190 ppb NH_3_, respectively, suggesting efficient sulfate formation on the Beijing haze particles. During the exposure process, the reaction rates $k_{{\mathrm{NO}}_{2}^{+}{\mathrm{HSO}}_{3}^{-}}$ at the two levels of NH_3_ are were 3.6 − 6.9 × 10^8^ M^−1^ s^−1^ (Tables S1 and S2), which are also 3–4 orders of magnitude larger the those for bulk solutions (46, 47) and in good agreement with the kinetics observed from the other seed experiments (Fig. 4), revealing that sulfate formation in the Beijing haze events accelerated at the air–aerosol aqueous interface via Eqs. 1 and 2. As a result, the molar ratio of ${\mathrm{SO}}_{4}^{2-}$ to SO_2_ at nighttime during the two haze episodes exponentially increased with an increasing HONO(g) concentration (Fig. 5D). These field evidences robustly show that the air–aerosol aqueous interface in China winter haze periods are dominated by anions due to the abundant chloride, nitrate, and carboxylic ions (Fig. 5B, Table S3), which accumulate at the air–aerosol aqueous interface and significantly enhance the uptake of NO_2_ and subsequent reaction with S(IV) at the aerosol surface. Recently, a few studies proposed that aqueous phase photochemistry including nitrate photolysis and oxalate-Fe(III) photochemistry could promote sulfate formation (48–50). Since those processes all proceed under strong irradiation conditions, which are thus not the cases for this study, because rapid sulfate formation in China haze periods always occurs under very weak solar radiation conditions and even at night (1, 10, 14, 15). Our recent study has compared the different formation pathways of sulfate in Beijing haze periods by using the kinetics of SO_2_ oxidation by NO_2_ obtained in this work, and found that SO_2_ oxidation by NO_2_ is the dominant formation pathway for sulfate in Beijing haze while others are relatively unimportant, in part due to low levels of oxidants such as O_3_ and H_2_O_2_ (7). Our current work also revealed that in the reaction process of NO_2_ with SO_2_, NO_2_ is reduced to HONO and the latter subsequently evaporates into the gas phase, which indicates that the aerosol-phase reaction is probably an important source of HONO in haze periods (17). HONO is a major source of OH radicals in the troposphere in some environments, and SO_2_ and NOx are abundant in many countries such as China and India. Thus, the enhancing effect of air–aqueous interface anions not only regulates atmospheric aerosol chemistry but also affects atmospheric oxidation capacity, which should be considered in future model simulations and other related studies.

## Materials and methods

Laboratory experiments were performed to evaluate SO_2_ oxidation by NO_2_ on various aerosols under dark conditions by using a home-made 1 m^3^ PTFE smog chamber (SI Appendix). To determine the interfacial effects of anions on the sulfate formation, we conducted chamber experiments by consecutively exposing different inorganic and organic particles to SO_2_, NO_2_, and NH_3_ under 90% RH conditions for ∼2.5 h and measuring the concentrations of the gas- and aerosol-phase components inside the chamber (see the details in SI Appendix S1) (1, 7, 17). The seeded particles are oxalic acid, NaCl, (NH_4_)_2_SO_4_, a NaCl and (NH_4_)_2_SO_4_ mixture, sucrose, NH_4_NO_3_ and water extracts of haze particles from Beijing, which are typical aerosols in Chinese haze periods. Field measurements of gaseous and PM pollutants were performed in Beijing from 2018 December 1 to 2019 January 31, by using the same instruments as those used for the laboratory smog chamber experiments. The sampling site is located on the rooftop (∼10 m above the ground) of a three-story building on the campus of the Chinese Research Academy of Environmental Science, which is located in the north part of Beijing city (SI Appendix S2). The pH values of particles inside the chamber and atmospheric PM_2.5_ in Beijing were estimated by utilizing the ISORROPIA-II model (SI Appendix S3). To illustrate difference in distributions of anions and cations at the air–aqueous interface and its effect on SO_2_ oxidation by NO_2_, we employed a MD model to simulate the ion distribution of Cl^−^ and Na^+^ at the air–water interface of 2.0 M NaCl solution droplets. Moreover, we also performed quantum chemical calculations to investigate the reaction pathway of SO_2_ with NO_2_ in the aqueous phase (see the details in SI Appendices S3 and S4).

## Supplementary Material

pgaf058_Supplementary_Data

## Data Availability

The chamber experiments and field observation data are available at https://doi.org/10.5281/zenodo.14898280, while all other data can be found at the Supplementary material.

## Preprints

This manuscript was posted as a preprint at: https://doi.org/10.21203/rs.3.rs-2230064/v1.
