# Supplementary material for: Atmospheric sulfate aerosol formation enhanced by interfacial anions
Source: PNAS Nexus. 2025 Feb 24;4(3):pgaf058. doi: 10.1093/pnasnexus/pgaf058 (PMC11880798; doi:10.1093/pnasnexus/pgaf058)
Supplement: pgaf058_Supplementary_Data [file pgaf058_supplementary_data.docx]

Supporting information for

**Atmospheric sulfate aerosol formation enhanced by interfacial anions**

Gehui Wang^1,^* ♀, Si Zhang^1^♀, Can Wu^1,^*, Tong Zhu^2^, Xinbei Xu^1^, Shuangshuang Ge^1^, Haitao Sun^3^, Zhenrong Sun^3^, Jiaxin Wang^4^, Yuemeng Ji^4^, Jian Gao^5^, Yanqin Ren^5^, Hong Li^5^, Fang Zhang^6^, Yuan Wang^7^, John H. Seinfeld^8^

^1^School of Geographic Sciences, Key Lab of Geographic Information Science of the Ministry of Education, East China Normal University, Shanghai 200241, China.

^2^School of Chemistry and Molecular Engineering, East China Normal University, Shanghai 200241, China.

^3^State Key Laboratory of Precision Spectroscopy, School of Physics and Electronic Sciences, East China Normal University, Shanghai 200241, China.

^4^School of Environmental Science and Engineering, Guangdong University of Technology, Guangzhou 510006, China.

^5^State Key Laboratory of Environmental Criteria and Risk Assessment, Chinese Research Academy of Environmental Sciences, Beijing 100012, China.

^6^School of Civil and Environmental Engineering, Harbin Institute of Technology (Shenzhen), Shenzhen 518055, China.

^7^Department of Earth System Science, Stanford University, Stanford, CA 94305, USA.

^8^Division of Chemistry and Chemical Engineering, California Institute of Technology, Pasadena, CA91125, USA.

♀These authors contributed equally to this work.

*Corresponding authors: Prof. Gehui Wang, E-mail: [ghwang@geo.ecnu.edu.cn](mailto:ghwang@geo.ecnu.edu.cn)

Dr. Can Wu, E-mail: [cwu@geo.ecnu.edu.cn](mailto:cwu@geo.ecnu.edu.cn);

**This PDF file includes:**

Supporting text S1 to S4

Figs. S1 to S7

Tables S1 to S6

References

**S1. Environmental Chamber Experiments**

In this study, a homemade Teflon environmental chamber with a size of 1.1m×0.8m×1.2m was used for the experiments (Fig. S1). We conducted chamber experiments by consecutively exposing different particles to SO_2_, NO_2_ and NH_3_ under 90% relative humidity (RH) conditions for approximately 2.5 hours and measuring the concentrations of the gas- and aerosol-phase components inside the chamber. The experimental procedures are briefly described as follows, and more details can be found in our previous studies (*1, 7, 17*).

1.1 Smog chamber experiment.

The smog chamber experiments were conducted in a zero air environment, which was produced by a Zero Air Supplier (Model 111 and Model 1150, Thermo Scientific, USA), while the smog chamber simulation experiments for the molecular formation pathway of SO_2_ oxidation were performed in a pure N_2_ environment because zero air has a small amount of N_2_O (approximately 50 ppb) and could influence the accuracy of the results. Relative humidity (RH) inside the chamber was adjusted by introducing a flow of humid vapor that was generated by bubbling zero air or pure N_2_ through ultrapure water (Milli-Q, 18.2 MΩ, Millipore Ltd., USA). The reaction temperature was kept constantly at 298 K. A hygrometer (Model 645, Testo AG, Germany) was used to measure RH during the experiments. To rule out the possible influence of TMI such as Fe^2+^ and Mn^2+^ on our chamber experiment results, 2mM ethylenediaminetetraacetic acid (EDTA) was added into the solution containing approximately 0.1% of the different seeds. Then the seed particles were generated and directly introduced into the chamber in a polydisperse mode through atomizing the solution by using a continuous flow aerosol particle generator (7388SJA, TSI Company, USA).

To investigate the role of air-aqueous interfacial anions in the sulfate formation process in China during haze periods, neutral and acidic inorganic and organic compounds, including NaCl, NH_4_NO_3_, (NH_4_)_2_SO_4_, a mixture of NaCl/(NH_4_)_2_SO_4_ (1:5, mass ratio), oxalic acid and sucrose, were chosen as seeds. Many field observations have reported that those compounds are typical components of atmospheric PM_2.5_ aerosols, especially in winter haze periods(*30, 51, 52*). Moreover, to further validate the interfacial anion effect on sulfate formation in haze periods in China, a polydisperse mode of seeds atomized from the water-extracts of atmospheric PM_2.5_ samples collected in Beijing during the 2018 winter haze events were also introduced into the chamber for reaction. The concentrations of those seeded particles inside the chamber were in the range of 1.0–5.0×10^4^ cm^-3^, depending on the seed type. The reaction gases NO_2_, SO_2_ and NH_3_, which were purchased from Air Liquide (China) Holding Co., Ltd., were consecutively injected into the reactor chamber by using a syringe. The initial concentrations of NO_2_ and SO_2_ were 600 ppb and 600 ppb, respectively, while the initial concentration of NH_3_ was 80 and190 ppb, respectively.

All the chamber experiments were conducted under dark conditions by fully covering the chamber with an anti-UV cloth hood. Prior to each experiment, the chamber was flushed with zero air or pure N_2_ (>99.999%) 10 times to ensure that the concentrations of the gaseous reactants and products and the particles inside the chamber were all below the detection limits of the instruments. The static electricity on the surface of the chamber was removed by using two ionizing air blowers near the chamber (*17, 53*).

A control experiment by exposing 2 mM EDTA seeds to NO_2_, SO_2_ and NH_3_, during which no detectable amount of sulfate was observed. Moreover, as seen in Figures S2D and S2E, during the experiments of (NH_4_)_2_SO_4_ and sucrose seeds no detectable amount of sulfate was observed. In contrast, all the other seeds showed a large amount of sulfate formed. Thus, the potential effect of the added EDTA on the SO_2_ oxidation can be neglected. Instead, the difference in sulfate production can only be attributed to the different properties of seeded particles.

1.2 Measurements on the chemical species in the chamber

(1) Gas-phase species of SO_2_(g), NO_X_(g) and HONO(g) andN_2_O(g)

The gas-phase NO_X_ (i.e., NO and NO_2_) and SO_2_ in the chamber were measured by a chemiluminescence nitrogen oxide analyzer (Ecotech EC9841), and a sulfur dioxide analyzer (Ecotech EC9852), respectively. N_2_O(g) and HONO(g) in the chamber were measured by using an isotopic N_2_O analyzer (LGR, Canada) and a commercial long path absorption photometer (LOPAP-03, QUMA, Germany), respectively. The detection limits were 1 ppt for N_2_O and 4.0 ppt for HONO. During the different reaction stages, 2 L of the gas in the chamber was collected by using an aluminum plastic composite membrane gas sample bag and analyzed for the concentration of N_2_O inside the chamber. To avoid interference from N_2_O in the air, the smog chamber and the sample bags were flushed at least 10 times by using high-purity N_2_ before each experiment and before gas sample collection to ensure that the concentration of background N_2_O in the chamber or the bags was below the instrument detection limit. The N_2_O analyzer was operated with the internal pump active (112 mL min^-1^) in slow mode (1 s) for approximately 10 min to obtain a steady baseline N_2_O concentration (*10, 54*). The collection efficiency of the LOPAP instrument for HONO is >99% with a time resolution of 30 s. The concentration of HONO was obtained by subtracting the interferences quantified in the second channel from the total signal obtained from the first channel. Hydrochloric acid (37%), sulfanilamide (>99%) and N-(1-naphthyl)-ethylenediamine dihydrochloride (>98%) used in this work were purchased from Sinopharm Chemical Reagent Co., Ltd, China.

(2) Aerosol-phase of SO_4_^2-^, NO_3_^-^ and NH_4_^+^.

Mass concentration and surface area of the polydisperse mode of aerosols in the chamber during the reaction course were continuously measured by a condensation particle counter (CPC, model 3762, TSI Company, USA), while SO_4_^2-^, NO_3_^-^ and NH_4_^+^in the chamber were continuously measured by a high-resolution time of flight aerosol mass spectrometer (HR-TOF-AMS, Aerodyne, USA).

1.3 Determination of the uptake coefficients of SO_2_ and NO_2_ during the sulfate formation process.

The uptake coefficient (γ) of SO_2_ oxidation by NO_2_ on the different seeded particles with various concentrations of NH_3_ can be calculated as follows:

|  | $\frac{\text{d[}\text{SO}_{\text{4}}^{\text{2-}}\text{]}}{\text{dt}}\text{=}\frac{\text{1}}{\text{4}}\text{γ}\text{ }\bar{\text{C}}\text{S[S}\text{O}_{\text{2}}\left( \text{g} \right)\text{]}$ | (E1) |
| --- | --- | --- |

where [SO_4_^2-^] is the molar concentration of sulfate during the reaction time period of dt. $\bar{\text{C}}$is the mean molecular speed of SO_2_ (370 m s^-1^), and S is the aerosol surface area, $\text{[}\text{S}\text{O}_{\text{2}}\left( \text{g} \right)\text{]}$ is the averaged molar concentration of SO_2_ in the gas phase during the whole reaction (*1, 2*).

|  | 2NO_2_ (g) +SO_2_ (g) + 2H_2_O + 2NH_3_ 🡪 2HONO(g) + SO_4_^2-^ + 2NH_4_^+^ (R1) |  |
| --- | --- | --- |

R1 is the overall aqueous phase reaction between SO_2_ and NO_2_ with neutralization by NH_3_ in the chamber (*1*). Stoichiometrically, the concentration of HONO (g) produced via the oxidation of SO_2_ by NO_2_ is two times the sulfate concentration. Thus, the uptake coefficients (γ) of NO_2_ by aerosols during the SO_2_ oxidation process can be calculated as follows:

|  | $\frac{\text{d}\left[ \text{HONO(g)} \right]}{\text{dt}}\text{=}\frac{\text{d(2×}\left[ \text{SO}_{\text{4}}^{\text{2-}} \right]\text{)}}{\text{dt}}\text{=K}\left[ \text{NO}_{\text{2}}\text{(g)} \right]\text{=}\frac{\text{1}}{\text{4}}\text{γ}\text{2}\bar{\text{C}}\text{S}\left[ \text{NO}_{\text{2}}\text{(g)} \right] (E3)$ | (E2) |
| --- | --- | --- |

where [HONO] and [sulfate] are the molar concentrations of HONO and sulfate produced by SO_2_ oxidation by NO_2_ during the reaction time period of dt, γ is the uptake coefficient of NO_2_, $\bar{\text{C}}$is the mean molecular speed of NO_2_ (411 m s^-1^), S is the aerosol surface area, and $[N\text{O}_{\text{2}}\left( \text{g} \right)\text{]}$ is the averaged molar concentration of NO_2_ in the gas phase during the whole reaction(*1, 2, 17*).

1.4 Correction for the concentration of ammonia in the chamber.

The loss of NH_3_ to the chamber wall is estimated by the first-order wall loss coefficient (k_w_) by considering gas-phase transport within the chamber according to Zhang et al (*55*).

|  | $\text{k}_{\text{w}}\text{=(}\frac{\text{A}}{\text{V}}\text{)}\frac{\text{α}_{\text{w}}\bar{\text{C}}}{\text{4+}\frac{\text{π}}{\text{2}}\text{(}\frac{\text{α}_{\text{w}}\bar{\text{C}}}{\sqrt{\text{k}_{\text{e}}\text{D}_{\text{g}}}}\text{)}}$ | (E3) |
| --- | --- | --- |

where A/V is the surface-to-volume ratio of the chamber, α_w_ is the mass accommodation coefficient of NH_3_ onto the Teflon chamber walls at RH = 90% (0.01) (*56*), $\bar{C}$ is the mean molecular speed of NH_3_ (603 m s^-1^) (*2*), k_e_ is the coefficient of eddy diffusion and is estimated to be 0.31 for the chamber used in this work, D_g_ is the gas-phase diffusion coefficient (1.98× 10^-5^) (*57*).

The average concentration of NH_3_ is estimated by E5,

|  | $\left[ \bar{\text{NH}_{\text{3}}} \right]\text{=}\frac{\text{1-}\text{e}^{\text{-}\text{k}_{\text{w}}\text{∆t}}}{\text{k}_{\text{w}}\text{∆t}}{\text{[}\text{NH}_{\text{3}}\text{]}}_{\text{0}}$ | (E4) |
| --- | --- | --- |

where the first-order wall-loss coefficient (k_w_) of NH_3_ in this study is 9.5 × 10^-3^ s^-1^ and Δt is the reaction time. For an initial injection concentration of 1000 ppb NH_3_, the average concentration of NH_3_ in the chamber was corrected to be 80 ppb.

1.5 Calculation on the reaction rate of SO_2_ with NO_2_

As seen in Tables S1 and S2, pH values of the wetted seed particles in the chamber are 3.5–5.0, which are similar to those observed in Beijing winter haze periods (Table S3). Under such moderate acidic conditions, NO_2_ reacts mostly with HSO_3_^-^ rather than SO_3_^2-^, as expressed by R1 (*2, 15, 46*).

2NO_2_(aq) + HSO_3_^-^ (aq) + H_2_O 🡪 H^+^(aq) + 2HONO(aq) + SO_4_^2-^(aq) (R1)

Thus, the overall formation rate of sulfate can be expressed as follows (*15, 46*):

d[SO_4_^2-^]/dt=$\text{k}_{\text{NO}_{\text{2}}\text{+}\text{HSO}_{\text{3}}^{\text{-}}}$×[HSO_3_^-^]× [NO_2_]_aq_ (E1)

Where d[SO_4_^2-^] is the molar concentration of sulfate produced during the reaction time of dt, *k*_NO2+HSO3-_ is the reaction rate constant of R1. [H^+^] is the molar concentration of hydrogen ions in the aqueous phase of seed particles, which was calculated by using the ISORROPIA-II model.

The partitioning of SO_2_ and NO_2_ gas into the aerosol aqueous phase can be estimated by using the Henry’s law (R2, R3). Moreover, SO_2_ in the aqueous phase dissociates into HSO_3_^-^ (R4), while NO_2_ does not dissociate.

SO_2_(g)↔ SO_2_•H_2_O, SO_2_•H_2_O= H_SO2_×P_SO2_ (R2)

NO_2_(g) ↔ NO_2_(aq), NO_2_(aq)=H_NO2_×P_NO2_  (R3)

SO_2_•H_2_O 🡪 H^+^+HSO_3_^-^ (R4)

Thus, [HSO_3_^-^]=K_a1_×SO_2_•H_2_O/[H^+^]=K_a1_×H_SO2_×P_SO2_/[H^+^], while [NO_2_]_aq_=H_NO2_×P_NO2_, in which K_a1_ is the thermodynamic dissociation constant of HSO_3_^-^$, \text{H}_{\text{SO}_{\text{2}}}$and $\text{H}_{\text{NO}_{\text{2}}}$are the Henry’s law constants of SO_2_ and NO_2_, $\text{P}_{\text{SO}_{\text{2}}}$and $\text{P}_{\text{NO}_{\text{2}}}$are the averaged partial pressures of SO_2_ and NO_2_ in the chamber during the reaction time of dt.

By combining R1-R4, the overall formation rate (E1) of sulfate can further be expressed as follows:

$$\frac{\text{d[}\text{SO}_{\text{4}}^{\text{2-}}\text{]}}{\text{dt}}\text{=}\left( \frac{\text{k}_{\text{NO}_{\text{2}}\text{+}\text{HSO}_{\text{3}}^{\text{-}}}}{\left[ \text{H}^{\text{+}} \right]} \right)\text{K}_{\text{a1}}\text{H}_{\text{SO}_{\text{2}}}\text{P}_{\text{SO}_{\text{2}}}\text{H}_{\text{NO}_{\text{2}}}\text{P}_{\text{NO}_{\text{2}}} (E2)$$

**S2. Field Measurements in Beijing**

Gaseous pollutants, including SO_2_(g), NO_2_(g) and O_3_(g), and the number concentration of ambient aerosols in Beijing were simultaneously measured from December 1, 2018, to January 31, 2019, by using the same instruments as those used for the laboratory smog chamber experiments. The detailed method was reported elsewhere (*1, 58*). Meteorological parameters (temperature and RH) were also monitored with a 1-hour time resolution. The sampling site is located on the rooftop (approximately 10 m above the ground) of a three-story building on the campus of the Chinese Research Academy of Environmental Sciences, which is situated in the northern part of Beijing city and near the 5^th^ ring highway.

The online mass concentration of PM_2.5_ was measured using an E-BAM (Met One Instruments, Inc., USA) system at a flow rate of 16.7 L min^-1^ with a PM_2.5_ inlet (BX-802, Met One, Inc., Grants Pass, OR, USA). Ambient NO_2_, SO_2_, and O_3_ were measured with a time resolution of 15 min by a chemiluminescence nitrogen oxide analyzer (Ecotech EC9841), sulfur dioxide analyzer (Ecotech EC9852) and ozone monitor (Ecotech EC9810), respectively (*59*). These 15-min data were averaged at a time resolution of 1 h and reported here.

Gas-phase NH_3_ and HONO were measured by a PICARO ammonia analyzer and a LOPAP. While aerosol-phase inorganic ions (i.e., SO_4_^2-^, NO_3_^-^, Cl^-^, NH_4_^+^, Ca^2+^, Na^+^ and K^+^) of PM_2.5_ were measured online with a PM_2.5_ inlet at a time resolution of 1 h by using a Monitor for AeRosols and GAses (MARGA, Metrohm Co., Switzerland) in ambient air. MARGA is a newly developed and widely used monitor for the in situ measurement of both gas- and aerosol-phase acidic and basic species(*60*). Rumsey et al (*61*) assessed the performance of the monitor under the US EPA Environmental Technology Verification (ETV) program and found that MARGA performed well in comparison to the denuder/filter pack for SO_2_, SO_4_^2−^, and NH_4_^+^, with all three compounds passing the accuracy and precision goals by a significant margin. In this study, we compared the hourly concentrations of SO_2_, SO_4_^2−^, and NH_4_^+^ from MARGA with those from the ultraluminescence method and filter-based measurements. The results showed good consistency between the two types of measurements (*1, 17*).

PM_2.5_ filter samples were collected on a day/night basis by using a high-volume PM_2.5_ sampler. The samples were collected onto a precombusted (450 °C, 8 hours) quartz fiber filter (Whatman 400, USA) at a flow rate of 1.13 m^3^ min^-1^. After collection, one-fourth of the quartz filter sample was cut into pieces and extracted with ultrapure water (>18 MΩ). Then, the water-extracted fraction was filtered to remove debris and divided into two parts. One part was analyzed by ion chromatography (IC) and a Shimadzu total organic carbon (TOC) analyzer for inorganic ions, oxalic acid and water-soluble organic carbon (WSOC), respectively (*62*). Another part was atomized into the smog chamber as a polydisperse mode of seeds for the chamber simulation experiments of SO_2_ oxidation.

**S3. Estimation of the Acidity (pH) of Particles in the Chamber and PM_2.5_ in Beijing**

The pH values of particles inside the chamber and atmospheric PM_2.5_ in Beijing were estimated by utilizing the ISORROPIA-II model, a subroutine commonly used in large-scale chemical transport models that incorporates both gas- and particle-phase measurements (*63, 64*). Accurate estimates of particle acidity can be determined to a high degree of accuracy on the basis of measurements of the semi-volatile partitioning of certain species (e.g., NH_3_/NH_4_^+^) (*63, 65-67*). The ISORROPIA-II thermodynamic model was used to estimate the equilibrium concentration of an aerosol composed of inorganic species (NH_4_^+^, Na^+^, K^+^, Mg^2+^, Ca^2+^, SO_4_^2-^, NO_3_^-^ and Cl^-^) and water. In this study, the ISORROPIA-II model was run in forward mode (i.e., incorporating gas and aerosol measurements) and metastable mode because winter haze pollution in Chinese cities, including Beijing, often occurs in very humid conditions with RH values greater than 80% (*1, 68-70*).

**S4. Molecular Dynamics and Quantum Chemical Calculations**

In this study, we employed a molecular dynamics (MD) model to simulate the ion distribution of Cl^-^ and Na^+^ at the air-water interface of 2.0 M NaCl solution droplets, of which the concentration was close to that of the seeded NaCl particles in the chamber under 90% RH conditions. Then, we performed quantum chemical calculations to investigate the reaction pathway of SO_2_ with NO_2_ in the aqueous phase (Tables S5 and S6).

4.1 Molecular dynamics simulation

MD simulations of sodium chloride solutions were performed in slab geometry with the OPENMM program (*71*). The unit cell contained 6113 water molecules, 130 sodium cations and chloride anions, which correspond to a 2.0 M NaCl salt concentration. The initial configurations of ions were obtained randomly in the water box. The size of the water box was 60.0 Å × 60.0 Å× 120.0 Å. The Drude force field was used to describe the intra- and intermolecular interactions in the system (*33*). The periodic boundary condition was employed. The simulation was run in the NVT ensemble for 200 ns, and the system temperature was kept at 298 K. The smooth particle mesh Ewald method was used to calculate the long-range electrostatic energies and forces, and the van der Waals interactions and the real space part of the Ewald sum were truncated at a distance of 12 Å(*72, 73*). The time step was 1 fs. The simulated trajectory was used to extract density profiles, i.e., the distributions of individual species in layers parallel to the interface, from the center of the slab across the air/solution interface into the gas phase (Fig. 2a). For the numerical evaluation of the density profiles, the z-axis was discretized to equidistant intervals of 0.5 Å. The frontier molecular orbitals (FMOs) of NO_2_ and Cl^-^ were obtained using density functional theory (DFT) at the B3LYP^2^/6-311++G(d,p) level by Gaussian 09 (*74*).

4.2 Quantum chemical calculations

To clarify the reaction pathway of SO_2_ with NO_2_ in the aerosol aqueous phase or the air-aqueous interface, we carried out a computational study to investigate the reactions of SO_2_ and NO_2_ in water by using density functional theory (DFT) and compare the NO_2_ disproportionation pathway with the redox between NO_2_ and SO_2_ (HSO_3_^-^) (Tables S5-S6). The calculations were performed with the Gaussian 09 program(*44, 74*). Unless otherwise mentioned, the geometry optimizations of the intermediates and transition states were performed at the PBE0/def-TZVPD level with D3-BJ dispersion correction (Tables S5 and S6); this level of calculation has been shown to be able to account for the difference in energy considered in this study. Solvation effects in water were accounted during optimization with the SMD method. The vibrational harmonic frequencies and thermal corrections were calculated using the same level as the optimization; the former confirmed that the optimized geometrical structures are the minima of PES and transition states (**TS**), the first-order saddle points. All energies mentioned here are the solvated Gibbs free energies in H_2_O (ΔG_sol_, ΔG_sol_^≠^). The long IRC trace for **TS1** was calculated with a lower double-$\zeta$ basis set of PBE0/ma-SVP (with SMD in water) due to the number of points. The starting point (IRC = 0) for IRC was reoptimized with a small step size limit at the same level of the IRC, with the PBE0/def-TZVPD level geometry as the initial guess. For this system, it was important to confirm that we had reached the correct electronic state. Specifically, we differentiated the [NO_2_^-^]···[HSO_3_^·^] state from the [NO_2_^·^] ···[HSO_3_^-^] state by performing population analysis on a long IRC path for transition state **TS1**. Fig. S7 shows the electronic energy, bond length change, and fragmented population analysis along the IRC path for the process of **IM1**🡪**TS1**🡪**IM2**. Judging by the bond length changes, we can see that the two hydrogen transfers happen in an asynchronous concerted way; in detail, the TS correlates to an H4 atom transfer, which then triggers a barrierless HAT of the H11 atom. The population analysis shows that before the reaction, in **IM1**, the electronic structure indeed correlates to NO_2_···HO(H) ···HSO_3_^-^, where the NO_2_ moiety has +0.91 spin population and -0.06 charge population, close to a pure NO_2_ radical, and the HSO_3_^-^ moiety has a +0.09 spin population and -0.94 charge population, close to a closed-shell HSO_3_^-^. Via **TS1**, **IM1** is smoothly connected to **IM2**, in which the electronic structure indeed correlates to HNO_2_···(H)OH···SO_3_^-^, where the NO_2_ moiety has a nearly +0.03 spin population, the HNO_2_ part has a -0.03 charge population, close to a closed-shell HNO_2_ molecule, and the SO_3_^-^ moiety has a +0.97 spin population and ‑0.99 charge population, close to an SO_3_^-^ radical. This suggests that **TS1** indeed reflects the barrier of the redox process.

**References:**

51. Z. J. Ding *et al.*, Summertime atmospheric dicarboxylic acids and related SOA in the background region of Yangtze River Delta, China: Implications for heterogeneous reaction of oxalic acid with sea salts. *Science of the Total Environment* **757**, (2021).

52. C. Yan *et al.*, Increasing contribution of nighttime nitrogen chemistry to wintertime haze formation in Beijing observed during COVID-19 lockdowns. *Nature Geoscience* **16**, 975-+ (2023).

53. C. Wu *et al.*, Efficient heterogeneous formation of ammonium nitrate on the saline mineral particle surface in the atmosphere of East Asia during dust storm periods. *Environmental science & technology* **54**, 15622-15630 (2020).

54. P. Han *et al.*, N_2_O and NOy production by the comammox bacterium *Nitrospira inopinata* in comparison with canonical ammonia oxidizers. *Water Research* **190**, 116728 (2021).

55. X. Zhang *et al.*, Influence of vapor wall loss in laboratory chambers on yields of secondary organic aerosol. *Proceedings of the National Academy of Sciences of the United States of America* **111**, 5802-5807 (2014).

56. A. Bongartz, S. Schweighoefer, C. Roose, U. Schurath, The mass accommodation coefficient of ammonia on water. *Journal of Atmospheric Chemistry* **20**, 35-58 (1995).

57. M. J. Tang, R. A. Cox, M. Kalberer, Compilation and evaluation of gas phase diffusion coefficients of reactive trace gases in the atmosphere: volume 1. Inorganic compounds. *Atmospheric Chemistry and Physics* **14**, 9233-9247 (2014).

58. S. Lv *et al.*, Gas-to-aerosol phase partitioning of atmospheric water-soluble organic compounds at a rural site of China: An enhancing effect of NH3 on SOA formation. *Environmental Science & Technology* **56**, (2022).

59. G. H. Wang *et al.*, Evolution of aerosol chemistry in Xi'an, inland China, during the dust storm period of 2013 &ndash; Part 1: Sources, chemical forms and formation mechanisms of nitrate and sulfate. *Atmos. Chem. Phys.* **14**, 11571-11585 (2014).

60. A. J. Ding *et al.*, Intense atmospheric pollution modifies weather: a case of mixed biomass burning with fossil fuel combustion pollution in eastern China. *Atmos. Chem. Phys.* **13**, 10545-10554 (2013).

61. I. C. Rumsey *et al.*, An assessment of the performance of the Monitor for AeRosols and GAses in ambient air (MARGA): a semi-continuous method for soluble compounds. *Atmos. Chem. Phys.* **14**, 5639-5658 (2014).

62. G. Wang *et al.*, Particle acidity and sulfate production during severe haze events in China cannot be reliably inferred by assuming a mixture of inorganic salts. *Atmos. Chem. Phys.* **18**, 10123-10132 (2018).

63. A. Nenes, S. N. Pandis, R. J. Weber, A. Russell, Aerosol pH and liquid water content determine when particulate matter is sensitive to ammonia and nitrate availability. *Atmos. Chem. Phys.* **20**, 3249-3258 (2020).

64. R. J. Weber, H. Guo, A. G. Russell, A. Nenes, High aerosol acidity despite declining atmospheric sulfate concentrations over the past 15 years. *Nature Geoscience* **9**, 282-285 (2016).

65. Y. N. Xie *et al.*, Nitrate-dominated PM2.5 and elevation of particle pH observed in urban Beijing during the winter of 2017. *Atmospheric Chemistry and Physics* **20**, 5019-5033 (2020).

66. H. Y. Guo *et al.*, Effectiveness of ammonia reduction on control of fine particle nitrate. *Atmospheric Chemistry and Physics* **18**, 12241-12256 (2018).

67. G. J. Zheng, H. Su, Y. F. Cheng, Role of Carbon Dioxide, Ammonia, and Organic Acids in Buffering Atmospheric Acidity: The Distinct Contribution in Clouds and Aerosols. *Environmental Science & Technology*, (2023).

68. M. A. Battaglia, R. J. Weber, A. Nenes, C. J. Hennigan, Effects of water-soluble organic carbon on aerosol pH. *Atmospheric Chemistry and Physics* **19**, 14607-14620 (2019).

69. S. J. Song *et al.*, Thermodynamic Modeling Suggests Declines in Water Uptake and Acidity of Inorganic Aerosols in Beijing Winter Haze Events during 2014/2015-2018/2019. *Environmental Science & Technology Letters* **6**, 752-760 (2019).

70. H. Q. Wang *et al.*, Model Simulations and Predictions of Hydroxymethanesulfonate (HMS) in the Beijing-Tianjin-Hebei Region, China: Roles of Aqueous Aerosols and Atmospheric Acidity. *Environmental Science & Technology* **58**, 1589-1600 (2023).

71. P. Eastman *et al.*, OpenMM 4: A Reusable, Extensible, Hardware Independent Library for High Performance Molecular Simulation. *Journal of Chemical Theory and Computation* **9**, 461-469 (2013).

72. J. Yang *et al.*, Unraveling a New Chemical Mechanism of Missing Sulfate Formation in Aerosol Haze: Gaseous NO2 with Aqueous HSO3-/SO32. *Journal of the American Chemical Society* **141**, 19312-19320 (2019).

73. K. Kholmurodov, W. Smith, K. Yasuoka, T. Darden, T. Ebisuzaki, A smooth-particle mesh Ewald method for DL_POLY molecular dynamics simulation package on the Fujitsu VPP700. *Journal of Computational Chemistry* **21**, 1187-1191 (2000).

74. M. J. Frisch *et al.,* I. Gaussian, Ed. (Wallingford, CT, USA, 2009).


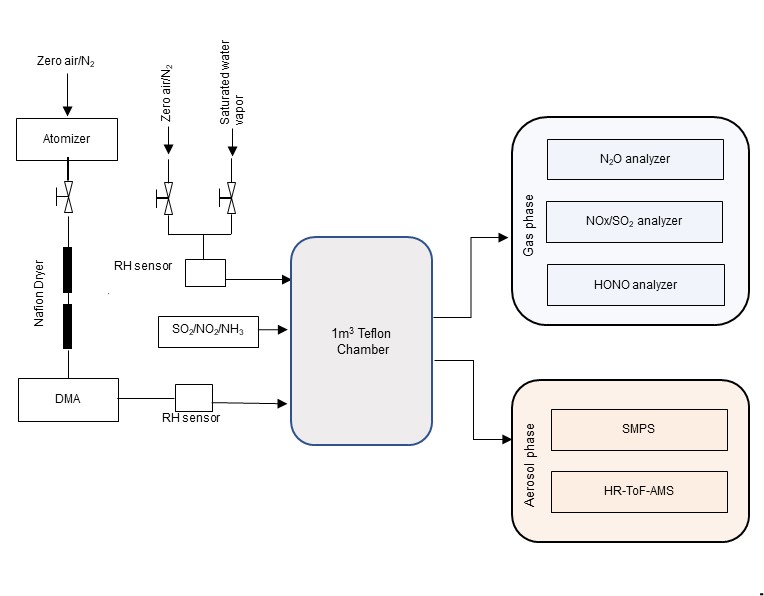


**Fig. S1. Schematic plot of the smog chamber experiment system**. DMA: differential mobility analyzer; HR-ToF-AMS: high-resolution-time of flight-aerosol mass spectrometry; SMPS: scanning mobility particle sizer; and TEM: transmission electronic microscopy.


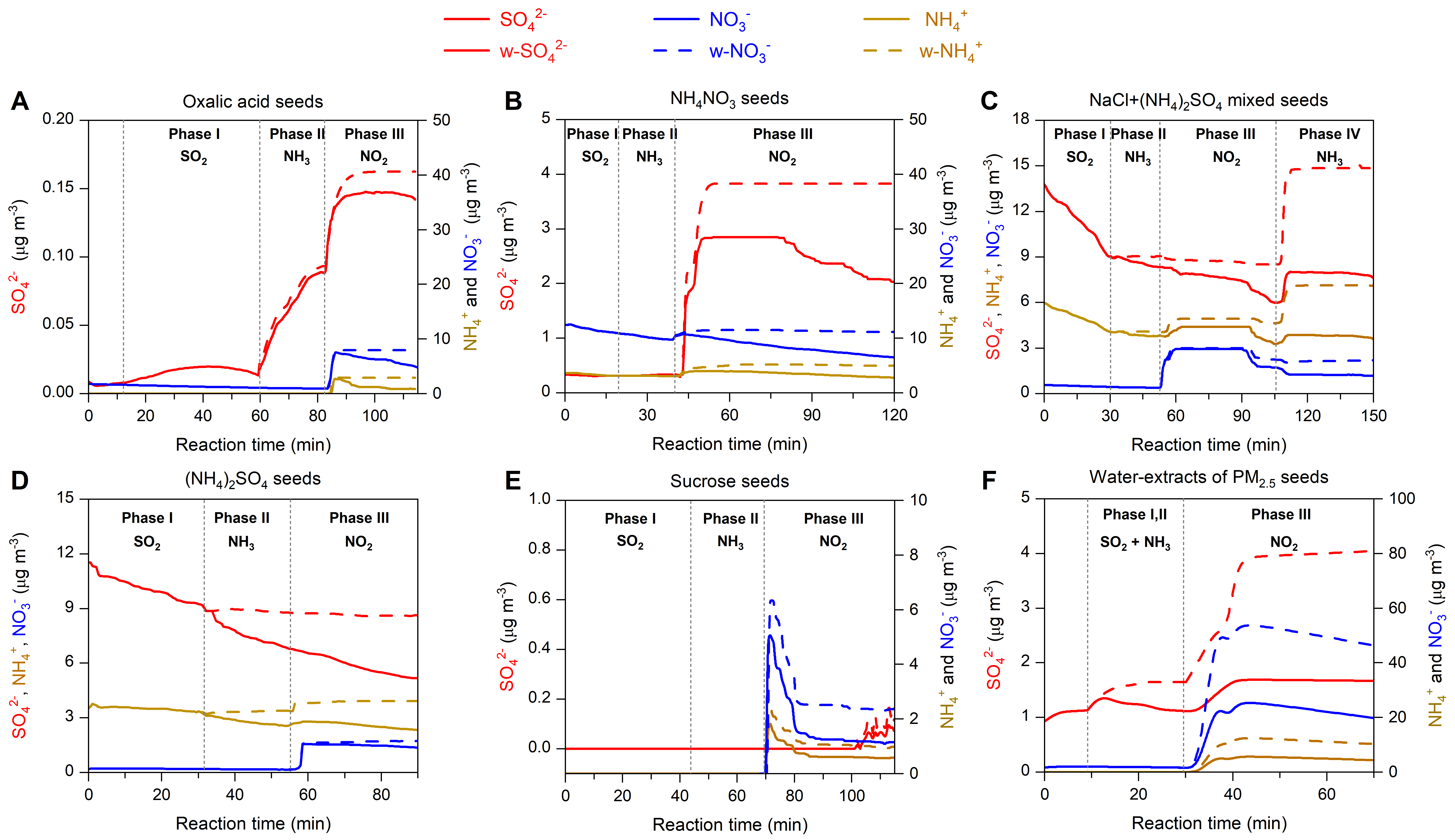


**Fig. S2. Changes in compositions of particles in the chamber during the exposures of different seeds to SO_2_ (600 ppb), NH_3_ (Phase II = 80 ppb, Phase IV=190 ppb) and NO_2_ (600 ppb) under 90%RH conditions.** The figure shows the time evolution of aerosol-phase species (SO_4_^2-^, NH_4_^+^_,_ NO_3_^-^, and wall-loss-corrected SO_4_^2-^ (w-SO_4_^2-^), NH_4_^+^(w-NH_4_^+^) and NO_3_^-^ (w-NO_3_^-^)) during the exposure experiments (w-SO_4_^2-^, w-NH_4_^+^ and w-NO_3_^-^ concentrations were derived by correcting SO_4_^2-^, NH_4_^+^, and NO_3_^-^ concentrations using a wall-loss rate before adding NH_3_ into the chamber. see Methods for more details).

**
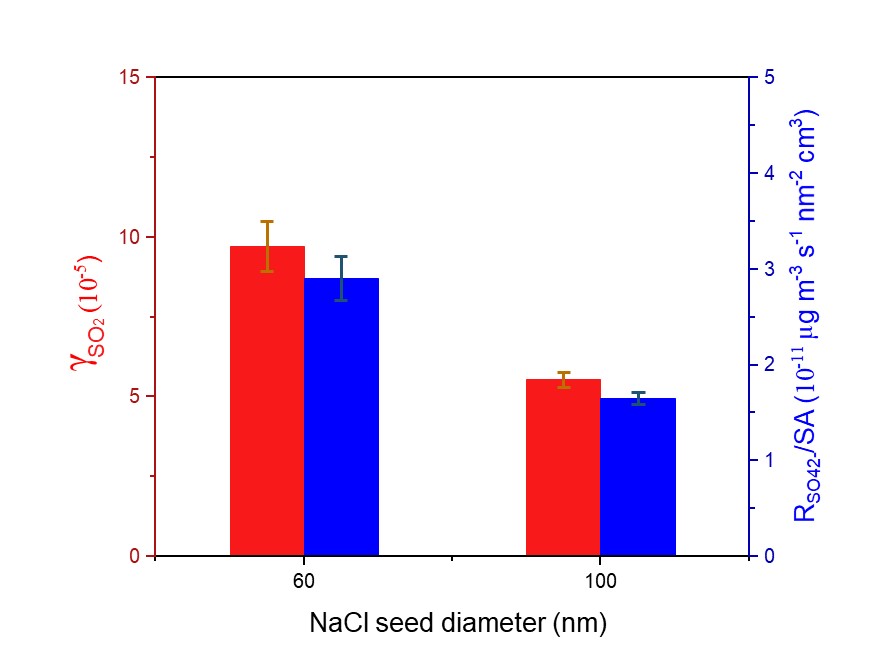
**

**Fig. S3. The dependence of sulfate production rate on the size of seed NaCl particles.** γ is the uptake coefficient of SO_2_ by NaCl seeds during the exposure to SO_2_, NH_3_ and NO_2_, while *R*_SO4_^2−^/SA is the sulfate formation rate (*R*_SO4_^2−^) normalized by the surface area (SA) of seeded NaCl particles in the chamber to eliminate the interference of the difference in initial concentrations.

**Fig. S4. Concentrations of gaseous and particulate species in the chamber as a function of reaction time during the exposure of NaCl seeds to NO_2_ (600 ppb), SO_2_ (600 ppb) and NH_3_ (190 ppb) under 90% RH conditions.** The concentration of HONO(g) inside the chamber was measured continuously during the experiment, while the concentrations of N_2_O, NO, NO_2_, SO_4_^2-^, NO_3_^-^ and NH_4_^+^ were measured after the HONO concentration in each phase had been constant for 20-30 min. The shaded part is the standard deviation.

**
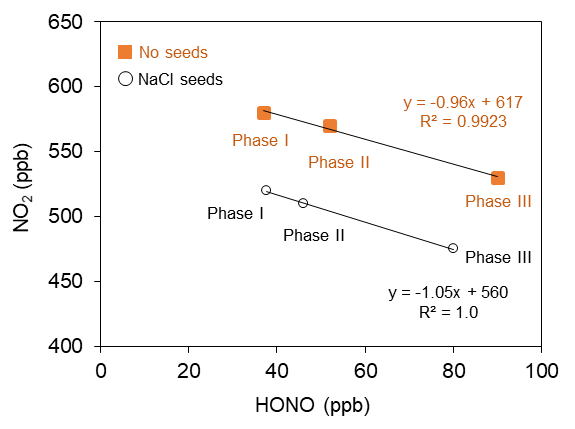
**

**Fig. S5.** **Concentrations of NO_2_ and HONO in the chamber after consecutively introducing NO_2_, SO_2_ and NH_3_ in the absence and presence of NaCl seeds under 90% RH conditions** (Phase I, 600 ppb NO_2_; Phase II, 600 ppb SO_2_; Phase III, 190 ppb NH_3_).


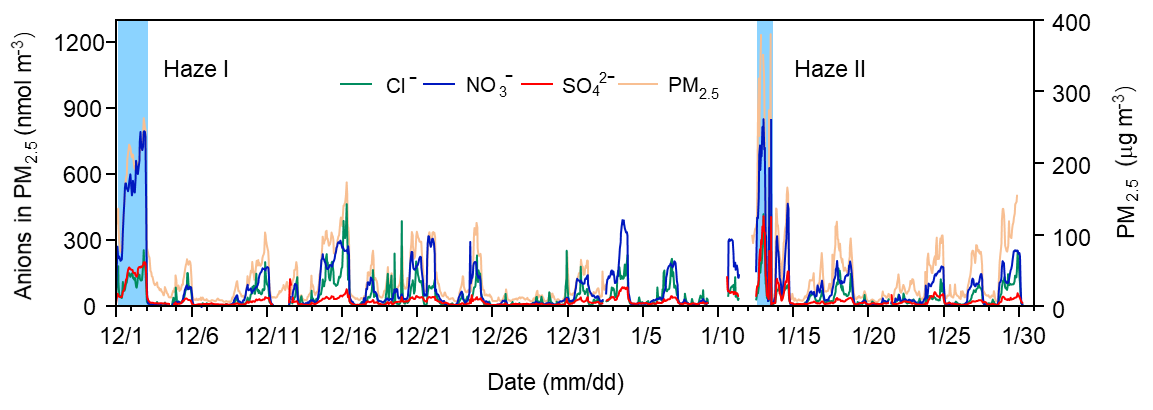


**Fig. S6.** **Temporal variations in molar concentrations of major anions (SO_4_^2-^, NO_3_^-^ and Cl^-^) of PM_2.5_ in Beijing during the 2018 winter campaign**. Blue shadows indicate a haze event with a daily PM_2.5_ larger than 200 μg m^-3^.

**
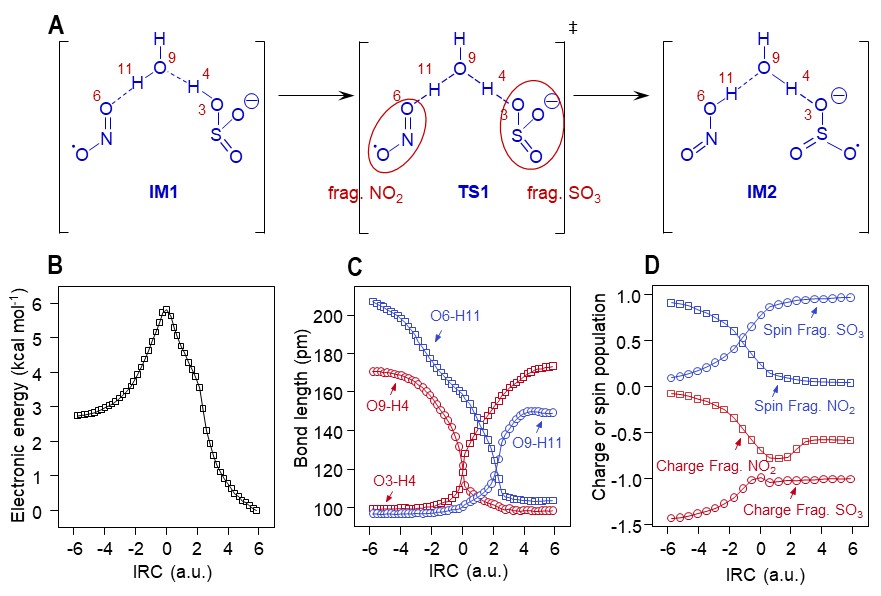
**

**Fig. S7.** **Electronic energy, bond length change, and fragmented population analysis along the IRC path of IM1🡪TS1🡪IM2.**

|  | **Table S1.** Uptake coefficients of SO_2_ and NO_2_ and reaction rate constant of NO_2_ with HSO_3_^-^ in the chamber during the exposure of the polydisperse mode of seeds to SO_2_, NO_2_ and 80 ppb NH_3_ at 293 K and 90% RH conditions. | | | | | | | | | | | | | | |
| --- | --- | --- | --- | --- | --- | --- | --- | --- | --- | --- | --- | --- | --- | --- | --- |
| Seeds | | P_SO2_ *^a^*  (atm) | P_NO2_ *^a^*  (atm) | H_SO2_  (M atm^-1^) | H_NO2_  (M atm^-1^) | SO_4_^2-^  (M) | γ_SO2_ | γ_NO2_ | K_a1_  (M) | Time  (s) | K_exp_  (M^-2^ s^-1^) | H^+^  (M) | pH | K_NO2+HSO3-_  (M^-1^ s^-1^) |  |
| NaCl | | 3.8×10^-7^ | 4.9×10^-7^ | 1.47 | 0.007 | 0.26 | 1.1×10^-5^ | 1.4×10^-5^ | 0.015 | 960 | 9.7×10^12^ | 1.1×10^-5^ | 5.0 | 1.1×10^8^ |  |
| (NH_4_)_2_SO_4_ | | 4.5×10^-7^ | 5.0×10^-7^ | 1.47 | 0.007 | / | / | / | 0.015 | / | / | 1.5×10^-4^ | 3.8 | / |  |
| (NH_4_)_2_SO_4_/NaCl | | 4.3×10^-7^ | 5.1×10^-7^ | 1.47 | 0.007 | / | / | / | 0.015 | / | / | 6.2×10^-5^ | 4.2 | / |  |
| Oxalic acid | | 5.6×10^-7^ | 4.5×10^-7^ | 1.47 | 0.007 | 0.02 | 3.7×10^-6^ | 8.3×10^-6^ | 0.015 | 600 | 9.2×10^11^ | 2.8×10^-4^ | 3.6 | 2.6×10^8^ |  |
| NH_4_NO_3_ | | 4.5×10^-7^ | 5.2×10^-7^ | 1.47 | 0.007 | 0.27 | 2.8×10^-6^ | 4.2×10^-6^ | 0.015 | 660 | 1.2×10^13^ | 7.2×10^-5^ | 4.1 | 8.5×10^8^ |  |
| Sucrose | | 4.0×10^-7^ | 4.7×10^-7^ | 1.47 | 0.007 | / | / | / | 0.015 | / | / | 3.3×10^-4^ | 3.5 | / |  |
| Water-extracts of Beijing-PM_2.5_ | | 5.0×10^-7^ | 5.2×10^-7^ | 1.47 | 0.007 | 0.13 | 2.0×10^-5^ | 3.4×10^-5^ | 0.015 | 720 | 4.5×10^12^ | 1.5×10^-4^ | 3.8 | 6.9×10^8^ |  |

*^a^* Average concentration of SO_2_ or NO_2_ in the chamber during the reaction.

|  | **Table S2.** Uptake coefficients of SO_2_ and NO_2_ and reaction rate constant of NO_2_ with HSO_3_^-^ in the chamber during the exposure of the polydisperse mode of seeds to SO_2_, NO_2_ and 190 ppb NH_3_ at 293 K and 90% RH conditions. | | | | | | | | | | | | | |  |
| --- | --- | --- | --- | --- | --- | --- | --- | --- | --- | --- | --- | --- | --- | --- | --- |
| Seeds | | P_SO2_ *^a^*  (atm) | P_NO2_ *^a^*  (atm) | H_SO2_  (M atm^-1^) | H_NO2_  (M atm^-1^) | SO_4_^2-^  (M) | γ_SO2_ | γ_NO2_ | K_a1_  (M) | Time  (s) | K_exp_  (M^-2^ s^-1^) | H^+^  (M) | pH | K_NO2+HSO3-_  (M^-1^ s^-1^) | |
| NaCl | | 2.8×10^-7^ | 2.8×10^-7^ | 1.47 | 0.007 | 0.91 | 2.2×10^-5^ | 4.0×10^-5^ | 0.015 | 1980 | 4.0×10^13^ | 2.4×10^-5^ | 4.6 | 9.7×10^8^ | |
| (NH_4_)_2_SO_4_ | | 2.5×10^-7^ | 3.7×10^-7^ | 1.47 | 0.007 | / | / | / | 0.015 | / | / | 7.4×10^-5^ | 4.1 | / | |
| (NH_4_)_2_SO_4_/NaCl | | 2.5×10^-7^ | 3.4×10^-7^ | 1.47 | 0.007 | 0.67 | 2.4×10^-6^ | 3.2×10^-6^ | 0.015 | 1200 | 4.5×10^13^ | 3.5×10^-5^ | 4.5 | 1.6×10^9^ | |
| Oxalic acid | | 3.0×10^-7^ | 3.0×10^-7^ | 1.47 | 0.007 | 0.14 | 1.2×10^-4^ | 2.1×10^-4^ | 0.015 | 1020 | 1.1×10^13^ | 9.2×10^-5^ | 4.0 | 9.7×10^8^ | |
| NH_4_NO_3_ | | 1.9×10^-7^ | 4.0×10^-7^ | 1.47 | 0.007 | 0.26 | 6.0×10^-6^ | 5.2×10^-6^ | 0.015 | 1020 | 2.2×10^13^ | 3.0×10^-5^ | 4.5 | 6.8×10^8^ | |
| Sucrose | | 2.2×10^-7^ | 3.2×10^-7^ | 1.47 | 0.007 | / | / | / | 0.015 | / | / | 2.7×10^-5^ | 4.6 | / | |
| Water-extracts of Beijing-PM_2.5_ | | 2.5×10^-7^ | 4.2×10^-7^ | 1.47 | 0.007 | 0.19 | 5.4×10^-5^ | 5.7×10^-5^ | 0.015 | 1020 | 1.2×10^13^ | 3.0×10^-5^ | 4.5 | 3.6×10^8^ | |

*^a^* Average concentration of SO_2_ or NO_2_ in the chamber during the reaction.

**Table S3**. Concentrations (μg m^-3^) of gaseous HONO, SO_2_ and NO_2_ and major components of PM_2.5_ in Beijing during 2018 winter.

|  | **Haze I** | | | |  | **Haze II** | | | |  | **Whole campaign** | | | |
| --- | --- | --- | --- | --- | --- | --- | --- | --- | --- | --- | --- | --- | --- | --- |
|  | Mean | Std | Min | Max |  | Mean | Std | Min | Max |  | Mean | Std | Min | Max |
| **I. Gaseous pollutants and meteorological parameters** | | | | | | | | | | | | | | |
| SO_2_ | 2.4 | 1.4 | 0.71 | 5.8 |  | 6.3 | 3.2 | 2.7 | 13 |  | 5.8 | 9.5 | ND*^a^* | 34 |
| NO_2_ | 41 | 5.8 | 30 | 50 |  | 75 | 16 | 41 | 92 |  | 21 | 19 | 6.9 | 92 |
| NH_3_ | 13 | 6.2 | 9.0 | 17 |  | 8.5 | 7.1 | 6.0 | 12 |  | 2.6 | 2.7 | ND*^a^* | 17 |
| HONO | 8.0 | 2.2 | 2.7 | 12 |  | 6.3 | 3.2 | 1.7 | 11 |  | 1.7 | 1.8 | ND*^a^* | 18 |
| RH (%) | 77 | 12.4 | 53 | 95 |  | 64 | 16 | 31 | 82 |  | 30 | 17 | 10 | 95 |
| T(^o^C) | 4.3 | 2.8 | -1.6 | 7.9 |  | -1.3 | 3.1 | -4.8 | 5 |  | -1.1 | 4.7 | -11.6 | 11.6 |
| **II. Major components of PM_2.5_** | | | | | | | | | | | | | | |
| Cl^-^ | 4.9 | 1.2 | 3.0 | 9.0 |  | 7.2 | 3.7 | 2.1 | 15 |  | 1.7 | 2.0 | ND*^a^* | 16 |
| NO_3_^-^ | 39 | 6.1 | 31 | 49 |  | 39 | 11 | 20 | 53 |  | 5.5 | 8.5 | ND*^a^* | 53 |
| SO_4_^2-^ | 16 | 2.0 | 11 | 20 |  | 24 | 9.9 | 8.6 | 39 |  | 2.8 | 3.8 | ND*^a^* | 39 |
| Na^+^ | 0.20 | 0.10 | 0.0 | 0.30 |  | 0.43 | 0.17 | 0.12 | 0.80 |  | 0.13 | 0.14 | ND*^a^* | 1.7 |
| NH_4_^+^ | 18 | 2.7 | 14 | 24 |  | 24 | 8.9 | 10 | 38 |  | 2.8 | 4.1 | ND*^a^* | 38 |
| K^+^ | 0.70 | 0.10 | 0.5 | 0.90 |  | 1.4 | 0.46 | 0.51 | 2.1 |  | 0.29 | 0.31 | ND*^a^* | 2.1 |
| Mg^2+^ | 0.10 | 0.04 | 0.0 | 0.10 |  | 0.25 | 0.07 | 0.14 | 0.40 |  | 0.10 | 0.08 | ND*^a^* | 0.61 |
| Ca^2+^ | 0.40 | 0.20 | 0.0 | 0.70 |  | 1.1 | 0.26 | 0.56 | 1.5 |  | 0.58 | 0.45 | ND*^a^* | 6.6 |
| total | 79 | 11 | 65 | 103 |  | 97 | 33 | 48 | 149 |  | 14 | 18 | ND*^a^* | 149 |
| PM_2.5_ | 214 | 21 | 179 | 263 |  | 274 | 94 | 71 | 379 |  | 44 | 51 | 5.0 | 379 |
| (Cl^-^+SNA)/PM_2.5_ | 0.35 | 0.03 | 0.33 | 0.40 |  | 0.37 | 0.14 | 0.17 | 0.67 |  | 0.24 | 0.10 | 0.04 | 0.69 |
| Cl^-^/ SO_4_^2-^ (mol/mol) | 0.84 | 0.13 | 0.64 | 1.3 |  | 0.78 | 0.11 | 0.67 | 1.0 |  | 1.6 | 1.4 | 0.0 | 13 |
| Cl^-^/ NO_3_^-^ (mol/mol) | 0.22 | 0.05 | 0.13 | 0.32 |  | 0.32 | 0.10 | 0.15 | 0.49 |  | 0.52 | 0.41 | 0.0 | 26 |
| total/PM_2.5_ | 0.37 | 0.03 | 0.33 | 0.41 |  | 0.37 | 0.14 | 0.17 | 0.69 |  | 0.27 | 0.11 | ND | 0.69 |
| pH*^b^* | 4.3 | 0.1 | 4.2 | 4.5 |  | 4.6 | 0.5 | 3.0 | 5.0 |  | 5.2 | 1.1 | 1.1 | 7.9 |
| ALWC*^b^* | 115 | 64 | 33 | 233 |  | 80 | 74 | 14 | 210 |  | 11 | 29 | 0.02 | 233 |
| Oxalic acid | - | - | - | - |  | 0.21 | 0.11 | 0.10 | 0.33 |  | 0.15 | 0.09 | 0.04 | 0.33 |

*^a^*ND: not detectable. *^b^*pH and ALWC (aerosol liquid water content) of PM_2.5_ were calculated by using ISORROPIA-II model.

**Table S4**. Uptake coefficient (γ) of SO_2_ on PM_2.5_ in Beijing during 2018 winter haze episodes.

|  | Average [SO_4_^2-^]  (μg m^-3^) | RH (%) | N (×10^4^) (cm^-3^) | Average D_p_ (nm) | S  (×10^-5^) (cm^2^cm^-3^) | SO_2_(g)  (μg m^-3^) | d[SO_4_^2-^]  (μg m^-3^) | dt  (hr) | γ_SO2_ |
| --- | --- | --- | --- | --- | --- | --- | --- | --- | --- |
| Haze I | 16 | 77 | 1.6 | 322 | 5.05 | 5.8 | 11.3 | 11 | (7.0 ± 1.2) ×10^-5^ |
| Haze II | 24 | 64 | 1.1 | 322 | 3.57 | 13 | 19.9 | 9 | (9.5 ± 0.2) ×10^-5^ |

**Table S5**. Geometries and energies of related compounds.

| **Species** | **IM1** | **TS1** | **IM2** | **TS-S1** | **IM-S2** | **IM3** | **TS3** | **IM4** | **IM5** |
| --- | --- | --- | --- | --- | --- | --- | --- | --- | --- |
| Optimization Level | PBE0-D3 (with SMD in water)/def2-TZVPD | | | | | | | | |
| Electronic Energy (kJ·mol^-1^) | -2377763.49 | -2377765.44 | -2377812.89 | -2377781.87 | -2377813.66 | -2376310.96 | -2376167.68 | -2376261.01 | -1076213.20 |
| Imaginaries (cm^-1^) | None*i* | 114.58*i* | None*i* | 182.60*i* | None*i* | None*i* | 451.99*i* | None*i* | None |
| H Correction (kJ·mol^-1^) | 178.85 | 171.37 | 181.00 | 178.98 | 184.84 | 152.86 | 142.97 | 156.61 | 75.24 |
| S (J·mol-1·K^-1^) | 454.45 | 431.31 | 478.29 | 416.23 | 409.37 | 421.28 | 387.79 | 427.97 | 336.50 |
| G Correction (kJ·mol^-1^) | 43.36 | 42.78 | 38.39 | 54.88 | 62.79 | 27.26 | 27.36 | 29.01 | -25.08 |
| Solvated Gibbs Free Energy (kJ·mol^-1^) | -2377963.84 | -2377962.55 | -2378009.41 | -2377972.85 | -2378014.46 | -2376514.52 | -2376474.37 | -2376531.52 | -1076265.40 |

**Table S5**. Geometries and energies of related compounds (Continued).

| **Species** | **IM6** | **TS4** | **IM7** | **H_2_O** | **HNO_2_** | **HNO_3_** | **HSO_4_^-^** | **N_2_O_4_** | **NO_2_** | **HSO_3_^-^** |
| --- | --- | --- | --- | --- | --- | --- | --- | --- | --- | --- |
| Optimization Level | PBE0-D3 (with SMD in water)/def2-TZVPD | | | | | | | | | |
| Electronic Energy (kJ·mol^-1^) | -1276768.20 | -1276762.27 | -1276821.50 | -200538.58 | -539749.97 | -737053.00 | -1836496.83 | -1076292.98 | -538111.01 | -1839558.91 |
| Imaginaries (cm^-1^) | None | 767.67*i* | None | None | None | None | None | None | None | None |
| H Correction (kJ·mol^-1^) | 147.97 | 136.59 | 151.16 | 65.59 | 64.97 | 81.08 | 86.71 | 78.85 | 33.67 | 141.53 |
| S (J·mol-1·K^-1^) | 389.12 | 372.37 | 389.63 | 188.71 | 246.50 | 265.88 | 293.58 | 309.02 | 239.45 | 372.13 |
| G Correction (kJ·mol^-1^) | 31.95 | 25.57 | 34.99 | 9.33 | -8.53 | 1.81 | -0.82 | -13.28 | -37.72 | 30.58 |
| Solvated Gibbs Free Energy (kJ·mol^-1^) | -1276795.16 | -1276785.21 | -1276805.14 | -200559.62 | -539770.39 | -737063.73 | -1836784.89 | -1076291.02 | -538135.21 | -1839860.84 |

**Table S6**. Optimized atomic coordinates.

| Element | X (Å) | Y (Å) | Z (Å) |
| --- | --- | --- | --- |
| N_2_O | | | |
| N | 0.884412 | 0.396489 | 0.000000 |
| O | 2.052537 | 0.579559 | 0.000000 |
| O | 0.193487 | -0.562933 | 0.000000 |
| H_2_O | | | |
| O | -0.355383 | -0.012904 | 0.000000 |
| H | 0.606536 | 0.031803 | 0.000000 |
| H | -0.634335 | 0.908764 | 0.000000 |
| HSO_3_^-^ | | | |
| S | -0.184179 | 0.011058 | -0.404453 |
| O | -0.675190 | 1.230904 | 0.287901 |
| O | 1.451029 | -0.021575 | 0.035779 |
| H | 1.864459 | 0.776838 | -0.323294 |
| O | -0.618011 | -1.215956 | 0.306860 |
| IM1 | | | |
| N | -2.190399 | -0.123690 | 0.012951 |
| N | -3.014662 | -1.639627 | 0.028780 |
| O | -2.609430 | 0.629866 | 0.815525 |
| O | -1.337060 | -0.077986 | -0.797803 |
| O | -3.867498 | -1.685112 | 0.839988 |
| O | -2.596138 | -2.393411 | -0.773877 |
| IM2 | | | |
| N | 1.768262 | 2.388352 | -0.057098 |
| O | 1.791678 | 2.973788 | -1.122148 |
| O | 1.685819 | 2.926655 | 1.028353 |
| O | 1.841971 | 1.086746 | -0.121623 |
| N | 1.697706 | 0.243732 | 1.542363 |
| O | 1.820895 | -0.819307 | 1.353515 |
| IM2^●●●^H_2_O | | | |
| N | 1.848438 | 2.527431 | -0.002619 |
| O | 1.635563 | 3.014142 | -1.106529 |
| O | 1.912160 | 3.235078 | 1.014024 |
| O | 2.013674 | 1.279604 | 0.095012 |
| N | 1.595485 | 0.240111 | 1.949835 |
| O | 2.127331 | -0.691549 | 1.855150 |
| O | 2.862551 | 1.658571 | 3.027818 |
| H | 2.352788 | 1.819525 | 3.832605 |
| H | 2.571807 | 2.354852 | 2.397088 |
| TS1 | | | |
| N | -1.529351 | -0.159867 | -0.004289 |
|  |  |  |  |
| **Table S6**. Optimized atomic coordinates (continued).  （c） | | | |
| Element | X (Å) | Y (Å) | Z (Å) |
| O | -2.730267 | -0.348635 | -0.036059 |
| O | -1.118399 | 1.065979 | -0.037367 |
| O | -0.700037 | -1.057217 | 0.054954 |
| N | 1.902599 | -0.276060 | -0.514004 |
| O | 2.588565 | -0.761159 | 0.202598 |
| O | 1.282280 | 1.208967 | 0.105973 |
| H | 1.535954 | 1.232771 | 1.044327 |
| H | 0.094154 | 1.103165 | 0.040585 |
| HONO | | | |
| N | 0.047374 | -0.015291 | -0.022016 |
| O | -0.042558 | -0.027880 | 1.337996 |
| H | 0.879194 | -0.015029 | 1.646945 |
| O | -1.015319 | -0.028533 | -0.520871 |
| HNO_3_ | | | |
| N | 2.083726 | 0.294713 | 0.049839 |
| O | 1.979291 | 0.809432 | 1.301120 |
| O | 2.296165 | -0.882604 | 0.018201 |
| O | 1.954788 | 1.054189 | -0.872762 |
| H | 1.807433 | 1.762294 | 1.178315 |
| TS2 | | | |
| S | -0.781303 | -0.015188 | 0.008574 |
| O | -1.030458 | 1.397405 | 0.185928 |
| O | -1.272145 | -0.881041 | 1.049263 |
| O | -0.946179 | -0.507005 | -1.339439 |
| N | 0.999068 | -0.079838 | 0.298802 |
| O | 1.572128 | -1.172166 | 0.300575 |
| O | 1.686529 | 0.928223 | -0.382522 |
| H | 1.208566 | 1.745666 | -0.162467 |
| IM4 | | | |
| S | -0.781303 | -0.015188 | 0.008574 |
| O | -1.030458 | 1.397405 | 0.185928 |
| O | -1.272145 | -0.881041 | 1.049263 |
| O | -0.946179 | -0.507005 | -1.339439 |
| N | 0.999068 | -0.079838 | 0.298802 |
| O | 1.572128 | -1.172166 | 0.300575 |
| O | 1.686529 | 0.928223 | -0.382522 |
| H | 1.208566 | 1.745666 | -0.162467 |
| IM5 | | | |
| S | 0.826479 | -0.022657 | -0.000020 |
| O | 1.182183 | -0.713553 | 1.216266 |
| O | 1.065179 | 1.398699 | 0.000484 |
| O | -0.861890 | -0.012747 | 0.000074 |
| **Table S6**. Optimized atomic coordinates (continued). | | | |
| Element | X (Å) | Y (Å) | Z (Å) |
| O | 1.182054 | -0.712670 | -1.216845 |
| N | -1.399314 | -1.313096 | -0.000370 |
| O | -2.561903 | -1.270676 | -0.000292 |
| HSO_4_^-^ | | | |
| S | -0.119316 | 0.021878 | -0.029098 |
| O | -0.503719 | -1.217871 | -0.672772 |
| O | -0.368784 | 0.054404 | 1.402862 |
| O | -0.580187 | 1.218534 | -0.714517 |
| O | 1.481662 | 0.001508 | -0.200407 |
| H | 1.857485 | 0.798881 | 0.201675 |
